# TC2N inhibits distant metastasis and stemness of breast cancer via blocking fatty acid synthesis

**DOI:** 10.1186/s12967-023-04721-3

**Published:** 2024-01-02

**Authors:** Xiang-lin Hao, Yang-fan Lv, De-feng Li, Fu-hai Bai, Ji Gong, Guang-qiang Pan, Lin-xi Su, Ya-li Wang, Wan-lei Fu, Bo Liu, Lu Huang, Dong Yan, Qiu-lin Tan, Jin-yi Liu, Qiao-nan Guo

**Affiliations:** 1 Department of Pathology, Xinqiao Hospital, Third Military Medical University, 183 Xinqiao Street, Shapingba District, Chongqing, 400037 People’s Republic of China; 2 Clinical Medical Research Center, Xinqiao Hospital, Third Military Medical University, Chongqing, 400037 People’s Republic of China; 3 Department of Anesthesiology, Xinqiao Hospital, Third Military Medical University, Chongqing, 400037 People’s Republic of China; 4 Institute of Toxicology, College of Preventive Medicine, Third Military Medical University, 30 Gaotanyan Street, Shapingba District, Chongqing, 400038 People’s Republic of China

**Keywords:** TC2N, Breast cancer, Metastasis, Cancer stem cell, Fatty synthesis, PTEN neddylation

## Abstract

**Background:**

Tandem C2 domains, nuclear (TC2N) is a C2 domain-containing protein that belongs to the carboxyl-terminal type (C-type) tandem C2 protein family, and acts as an oncogenic driver in several cancers. Previously, we preliminarily reported that TC2N mediates the PI3K-Akt signaling pathway to inhibit tumor growth of breast cancer (BC) cells. Beyond that, its precise biological functions and detailed molecular mechanisms in BC development and progression are not fully understood.

**Methods:**

Tumor tissues of 212 BC patients were subjected to tissue microarray and further assessed the associations of TC2N expression with pathological parameters and FASN expression. The protein levels of TC2N and FASN in cell lines and tumor specimens were monitored by qRT-PCR, WB, immunofluorescence and immunohistochemistry. In vitro cell assays, in vivo nude mice model was used to assess the effect of TC2N ectopic expression on tumor metastasis and stemness of breast cancer cells. The downstream signaling pathway or target molecule of TC2N was mined using a combination of transcriptomics, proteomics and lipidomics, and the underlying mechanism was explored by WB and co-IP assays.

**Results:**

Here, we found that the expression of TC2N remarkedly silenced in metastatic and poorly differentiated tumors. Function-wide, TC2N strongly inhibits tumor metastasis and stem-like properties of BC via inhibition of fatty acid synthesis. Mechanism-wise, TC2N blocks neddylated PTEN-mediated FASN stabilization by a dual mechanism. The C2B domain is crucial for nuclear localization of TC2N, further consolidating the TRIM21-mediated ubiquitylation and degradation of FASN by competing with neddylated PTEN for binding to FASN in nucleus. On the other hand, cytoplasmic TC2N interacts with import proteins, thereby restraining nuclear import of PTEN to decrease neddylated PTEN level.

**Conclusions:**

Altogether, we demonstrate a previously unidentified role and mechanism of TC2N in regulation of lipid metabolism and PTEN neddylation, providing a potential therapeutic target for anti-cancer.

**Supplementary Information:**

The online version contains supplementary material available at 10.1186/s12967-023-04721-3.

## Background

Worldwide, the incidence of breast cancer (BC) has surpassed the lung cancer to occupy the first position in malignant tumors [1]. Despite advances in BC pathophysiology, diagnosis, prognosis and therapy, distant metastasis remains a leading cause of death and decreasing life expectancy [2]. Tumor metastasis, which is represented by cancer cell spreads to farther tissues/organs from primary lesions through the blood or lymphatic system, have been regarded as a complex, multistage process [3]. Despite the fact that our understandings of this complex multistep process are expanding, the underlying molecular mechanisms of BC metastasis remains obscure, and their understanding contribute to develop more effective therapies and improve the prognosis [2]. Intratumoral heterogeneity is another major reason for the poor survival [4], this phenotypic diversity seems to be caused mainly by cancer stem cells (CSCs), which are a small population of tumor cells that exhibit the self‐renewal capacity and are responsible for tumor initiation and progression [5]. Although the mechanisms of CSCs maintenance have been explored to a great extent, the key genes that regulate CSCs in BC, are still not entirely clear.

The C2 domain is an evolutionarily conserved domain, which consists of about 130 residues [6]. A large and diverse set of eukaryotic proteins containing C2 domain have been confirmed to be a key player of tumorigenesis and progression. For instance, Smurf1 is involved in regulation of lung cancer cell growth and migration through directly interacting with the kinase domain of PIPKIγ by its C2 domain [7]. Myoferlin facilitates metastasis by regulating cellular lipid metabolism in triple-negative BC [8]. CC2D1A promotes the acquisition of chemotherapy resistance in ovarian cancer [9]. Moreover, the C2 domain of PTEN and PI3KCA are necessary for their molecular function, and may be a potential biomarker or target in cancer [10, 11].

In our previous studies, we have identified a C2 domain-containing protein TC2N in lung cancer, and further uncovered its expression, biological function and underlying molecular mechanisms in regulation of tumor growth and metastasis [12, 13]. Subsequently, the prognostic significance and pivotal role of TC2N in many cancers have been disclosed [14–17]. Our later study found that TC2N has a opposite function in BC, its act as a tumor suppressor by inhibiting tumor growth via PI3K-Akt signaling [18]. However, its other function and mechanisms of involvement in BC-related processes insufficiently elucidated. Considering the vital and unique role of TC2N in BC, it is indispensable to deep understanding of its function, mechanisms and clinicopathological significance.

In this study, we have identified TC2N as a tumor metastasis and stemness-related gene in BC. Analyzing the clinicopathologic significance of TC2N, we found TC2N expression is related to metastasis status and tumor differentiating degree of patients. The decisive role of lipid metabolism in tumor progression and metastasis have been emphasized [19, 20]. Importantly, we here report TC2N to be a tumor suppressor that blocks fatty acid synthesis through preventing neddylated PTEN-mediated FASN stabilization. Our findings provide a novel function and mechanism in TC2N-mediated tumor progression.

## Methods

### Cell culture

The human breast carcinoma cell lines MDA-MB-231 (M231) MCF7, BT474 and SKBR3 were obtained from the CBCAS (Cell Bank of Chinese Academy of Science, Shanghai, China). The E0771 murine breast carcinoma cell line was purchased from Hunan Fenghui Biotechnology Co., Ltd (Hunan, China). BC cells with TC2N stable expression or TC2N knockdown were reutilized as described in our previous study [18]. These cell lines were cultured in DMEM media (HyClone, Logan, UT, USA) supplemented with 10% fetal bovine serum (FBS, HyClone), and were incubated in 5% CO_2_ at 37 °C. The cells were treated with 0.2 μM TVB-3166 (Sigma-Aldrich, USA) for 48 h to block FASN activity.

### Patient samples and tissue microarray (TMA) construction

A total of 212 BC samples were collected from Xinqiao Hospital Affiliated to Third Military Medical University (Chongqing, China) from January, 2017 to December, 2021. Clinical and pathological information was retrieved from the patients’ electronic medical records (Additional file 1: Table S1). Tumor staging was performed according to the TNM system of the American Joint Committee on Cancer (AJCC) [21]. The Nottingham system was used to determine the tumor grade [22]. The collection of tumor specimens and clinicopathological information was approved by the Ethics Committees in Xinqiao Hospital Affiliated to Third Military Medical University (Chongqing, China). Written-informed consent was obtained before the investigation. All procedures were conducted according to the provisions of the Helsinki Declaration in 1975. For tissue microarray construction, all specimens were fixed in 4% paraformaldehyde fix solution and then paraffin-embedded. The TMA contains 212 tumor tissues were produced by tissue microarray instrumentation as previously described [23].

### Mice

BALB/c nude mice (6-week-old, female) were purchased from Vital River Laboratories Co., Ltd (Beijing, China). TC2N knockout mice (C57BL/6, 6-week-old, female) were generated by a commercial supplier (Cyagen Biosciences, Guangzhou, China) using the CRISPR-Cas9 technique. All mice maintained in a controlled environment (12-h light/dark cycle, ad libitum access to food and water). Mice experiments conformed to the Guide for Care and Use of Laboratory Animals, and were approved by the Ethics Committees in Xinqiao Hospital, Third Military Medical University.

For the tumor metastasis assay, the mice (n = 8, sum) were randomly divided into two groups (n = 4 per group), Vc and TC2N. The stably transfected SKBR3 cells were digested, collected and suspended in PBS. The suspended cells were injected into 6-week-old Balb/c female nude mice by the left ventricle at 2 × 10^6^ cells/mouse. After 50 days housing, the mice were sacrificed, and the tissues were dissected for tissue observation (H&E staining).

For the tumor growth assay, the female nude mice (n = 10, sum) were randomly divided into two groups (n = 5 per group), Vc and TC2N. The stably transfected M231-spheres were treated as described above and then injected into the right flanks of 6-week-old Balb/c nude mice at 2 × 10^6^ cells/mouse. After 29 days housing, the mice (with a maximum tumor diameter stretching 1.5 cm) were sacrificed, and tumor tissues were exfoliated for tissue observation and weighing.

For the observation of fatty deposition, the mice (n = 8, sum) were randomly divided into two groups (n = 4 per group), Vc and TC2N. The stably transfected E0771 cells were transplanted into the bilateral mammary fat pads of 6-week-old Balb/c female nude mice at 2 × 10^6^ cells/mouse. After 21 days housing, the mice were sacrificed, and tumor tissues were exfoliated for tissue observation and oil red O staining.

TC2N knock-out mouse model and genotype identification. C57BL/6J (TC2N-knockout) engineered mice were generated through deletion of exon 3–8 of TC2N (ENSMUST00000162735.7) by a commercial supplier (Cyagen Biosciences, Santa Clara, CA, USA) using the CRISPR-Cas9 technique. Mice were maintained in a controlled environment (12-h light/dark cycle, ad libitum access to food and water). The genotype of the mice was determined by RT-PCR (PCR procedure: pre-denaturation at 95 °C for 5 min; 20 cycles of 98 °C for 30 s, 65 °C for 30 s with decreasing 0.5 °C each cycle, 72 °C for 45 s; 20 cycles of 98 °C for 30 s, 55 °C for 30 s, 72 °C for 45 s and extension of 5 min at 72 °C) and the primer sequences were listed as follows: Tc2n-KO-F- TATAGAAGCTCAGGCAGAGGCAG, Tc2n-KO-R- GGATGAGGGTCAGAGACACTAATG, Tc2n-wt-F- CCTGATTTCCAGGTGTGTTAGTG and Tc2n-wt-R- GACTCAACAAGCTGAAGAACTCCC. The PCR products were performed by electrophoresis as previously described [24]. For the assessment of tumor formation in TC2N-knockout mice, the TC2N +/+ (W/W) mice (n = 4) and TC2N−/− (K/K) mice (n = 4) that were matched for age (14 weeks) and weight (16–19 g), were used. Murine E0771 breast carcinoma cells were injected into injected into the mammary fat pad of these mice (W/W and K/K) at 2 × 10^6^ cells/mouse. Mice were sacrificed at 21 days after injection, and tumors were examined by tissue observation.

### Plasmids, lentivirus, shRNA and transfection

The construction of shRNA expression vector, the full-length open reading frame of TC2N and truncation lentivirus were performed as previously described [12, 18]. For overexpression of murine TC2N, the full-length open reading frame of murine TC2N was synthesized and constructed into pIRES2-EGFP expression vector (Invitrogen Preservation, Carlsbad, CA, USA). 2 μg plasmids of TC2N or vector control were transfected into E0771 cells for 48 h using 6μL Lipofectamine 2000 Reagent (Invitrogen) and screened under G418 (Calbiochem, La Jolla, CA, USA). For human TC2N knockdown, 2 μg oligomeric single-stranded oligonucleotides (shRNA) that were used in our previously studies [12, 13, 18] were transfected into SKBR3 cells using 6 μl Lipofectamine 2000 Reagent (Invitrogen) for 48 h and screened under puromycin (Beyotime, Shanghai, China). Cell clones were obtained by the cylinder method.

### The public databases

The RNA-Seq data of all 1098 BC patients and protein data of all 134 BC patients were directly downloaded from TCGA (https://www.cancer.gov/) and Clinical Proteomic Tumor Analysis Consortium (CPTAC) (https://proteomics.cancer.gov/data-portal) databases, respectively. The RNA expression profiles from GSE102484, GSE93601, GSE48091, GSE25307, GSE58212, GSE20685, GSE86166, GSE62931, GSE80999, GSE27830, GSE22219, GSE19783, GSE26639, GSE36774, GSE76275, GSE59590, GSE31448, GSE167213, GSE162228, GSE59198, GSE73235, GSE36771, GSE146558, GSE88770, GSE42568 and GSE37181 datasets were downloaded from GEO database (https://www.ncbi.nlm.nih.gov/geo/).

### Gene ontology (GO) analysis

For GO analysis, the RNA-Seq data from TCGA and GEO database were used to analyze the correlation between TC2N expression and all other genes in BC patients. The protein data from CPTAC database was used to analyze the correlation between TC2N expression and all other proteins in BC patients. The R project was used to screen co-expressed genes or proteins of TC2N based on above public data. All co-expressed genes or proteins significantly correlated with TC2N expression were used for GO or Reactome pathway analyses by using Gene Ontology Resource (http://geneontology.org/). Specifically, log in the website and input co-expressed genes or proteins into the textbox then click “launch” to proceed to the next page. Next, in the “Annotation Data Set” section, select “Reactome pathways” for data type and click “launch analysis” for getting GO results. The R code is added as Additional file 2: Data S1 and co-expressed genes and proteins of TC2N are listed in Additional file 3: Data S2.

### Gene set enrichment analysis (GSEA)

GSEA is a computational method that determines whether an a priori defined set of genes shows statistically significant, concordant differences between two biologic states. The GSEA software (v4.3.2) was obtained from the GSEA official website (https://www.gsea-msigdb.org/gsea/index.jsp). Specifically, the RNA-Seq data was converted to a “gct” file (gene.gct) and then uploaded to the “Load data” section for further analysis. In the next “Run GSEA” section, the parameters were established, such as gene set database: C2.cp.Reactome v6.2.symbols.gmt (curated); number of permutations: 1,000; Phenotype labels: use TC2N as a phenotype; P value < 0.05 and a false discovery rate (FDR) < 0.25 were considered as meaningful. The statistical parameter of these biological processes and signals are listed in Additional file 4: Data S3.

### H&E staining and immunohistochemical (IHC)

Formalin-fixed paraffin-embedded specimens were sectioned at 5 μm for H&E staining or IHC. The sections were dewaxed three times in xylene and sequentially rehydrated in baths of decreasing methanol content (100/95/70/50%), and followed by incubation with 3% H_2_O_2_ in 90% methanol for 30 min to ensure neutralization of endogenous peroxidase. The slides were stained with hematoxylin and eosin for histological analysis. IHC was performed as described previously [25]. Briefly, the protein expression of TC2N and FASN were detected using their primary antibodies: 1:200 diluted rabbit polyclonal antibody (Cat. #: HPA027549; Sigma) for TC2N; 1:500 diluted rabbit polyclonal antibody (Cat. #: 10624-2-AP; Proteintech) for FASN. A combined score of intensity and distribution was used to categorize the immunohistochemical staining for these proteins, as previously described by us and others [12, 26].

### Cell immunofluorescence

For immunofluorescence, cell slides were fixed with 4% paraformaldehyde, permeated in 0.3% Triton X-100 (PBS), blocked with 3% goat serum for 30 min, and stained using the indicated antibodies. After incubation with primary antibodies for 16 h at 4 °C, the slides were then incubated with goat anti-rabbit/mouse IgG H&L Alexa Fluor 488 or 594 antibody for 1 h at 37 °C, respectively. The nuclei were stained with DAPI, and images were acquired with an inverted confocal microscope (Leica, Mannheim, Germany).

### Total fatty acids analysis

BC cells were collected and centrifuged at 3000 rpm for 10 min. After centrifugation, cell pellets were lysed in extracting buffer on ice for 30 min and then centrifuged at 8000 rpm for 10 min for further analysis. Total fatty acids level of BC cells was determined by using fatty acid detection kit (Solarbio, Beijing, China) according to the manufacturer’s protocol. Briefly, the cell pellets were collected and subjected to ice-cold lysis using extraction buffer for 30 min. After centrifugation at 4 °C, 8000 rpm for 10 min, the supernatant was collected, and the absorbance of the samples was measured at 550 nm. Subsequently, the concentration of free fatty acids in the cell samples was calculated based on a standard curve.

### Lipidomics analysis

The analysis of fatty acid composition was performed by Novogene Co, Ltd, (Beijing, China). For lipid extraction, about 5 mg total protein of M231 cells was mixed with 0.75 ml methanol and 2.5 ml methyltert-butylether, and the mixture was incubated for 1.5 h at room temperature in a shaker. Then, 0.625 ml of MS-grade water was added and mixed at room temperature for phase separation. Upon 10 min of incubation, the mixture was centrifuged at 1000 g for 10 min and the upper phase was collected, and the lower phase was re-extracted with 1 mL of the solvent mixture (methyltert-butyl ether/methanol/water (10:3:2.5, v/v/v)), and the upper phase was collected again. Combined upper phases were dried and dissolved in 100 μL of isopropanol for further UHPLC-LC–MS/MS analysis. A Vanquish UHPLC system (Thermo Fisher, Germany) coupled with an Orbitrap Q ExactiveTM HF mass spectrometer (Thermo Fisher, Germany) were utilized to perform UHPLC-MS/MS analyses. For analysis of fatty acid composition, above samples were injected into an accucore column (Thermo Fisher, Germany) using a 20-min linear gradient at a flow rate of 0.35 ml/min. Flowing phase was prepared as follows: buffer A (acetonitrile/water (6/4), 10 mM ammonium acetate and 0.1% formic acid), buffer B (acetonitrile/isopropanol (1/9), 10 mM ammonium acetate and 0.1% formic acid). The solvent gradient was set as follows: 70% buffer A/30% buffer B, initial; 70% buffer A/30% buffer B, 2 min; 57% buffer A/43% buffer B, 5 min; 45% buffer A/55% buffer B, 5.1 min; 30% buffer A/70% buffer B, 11 min; 1% buffer A/99% buffer B, 16 min;70% buffer A/30% buffer B, 18.1 min. For data analysis, the Compound Discoverer 3.01 (CD3.1, Thermo Fisher) was used to analyze the raw files of each metabolite that generated by UHPLC-MS/MS and perform peak alignment, peak picking, and quantitation. After normalization of these peak intensities, the normalized data was matched with the Lipidmaps and Lipidblast database to obtained the accurate qualitative and relative quantitative results. These data were expressed as μmol of fatty acids per gram of sample, which are listed in Additional file 5: Data S4.

### RNA extraction and RT-qPCR analysis

Total RNAs were extracted from M231 and MCF7 cells according to the manual instructions of Trizol (Invitrogen, Carlsbad, CA, USA). The RNAs were quantified and qualified with Agilent 2100 bioanalyzer and Nano Drop (Thermo Fisher Scientific, MA, USA). The ribosomal RNA (rRNA) was removed including 5S rRNA, 5.8S rRNA, 18S rRNA, 28S rRNA, 12S rRNA and 16S rRNA from the RNAs. The RNAs were then fragmented into small pieces, and the cleaved RNA fragments were copied into first strand cDNA, followed by second strand cDNA synthesis. We used the SYBR Premix Ex Taq II (Takara, Dalian,China) and an CFX96™ Real-Time PCR Detection System (Bio-Rad Laboratories) to perform the quantitative real-time PCR (qRT–PCR) to determine the quantitative expression of genes. The 2^−ΔΔct^ method was used to calculate the relative expression levels of genes. The primers synthesized by Tsingke Biotechnology (Beijing, China), and the sequence of primers are listed in Additional file 6: Table S2.

### Protein extraction and WB analysis

Cells or tissues were lysed in SDS lysis buffer (Beyotime, Shanghai, China) supplemented with PMSF (Beyotime, Shanghai, China) to extract total protein. The BCA quantification kit (Beyotime, Shanghai, China) was used to perform protein quantification. Twenty micrograms of protein were mixed with loading buffer (Beyotime, Shanghai, China) and denatured for 5 min at 100 °C. The cytoplasmic and nuclear extracts of cells were obtained using nuclear and cytoplasmic protein extraction kit (Beyotime, Shanghai, China) as previously described [27].

The western blotting (WB) analysis was performed to determine protein expression. Thirty micrograms of protein were run on 6% or 10% sodium dodecyl sulfate–polyacrylamide gel electrophoresis. The proteins on sodium dodecyl sulfate–polyacrylamide gel was transferred to polyvinylidene difluoride membrane (Millipore Corporation, Bedford, MA, USA). The membranes blocking with 5% milk for 2 h at room temperature with primary antibodies was incubated in incubator overnight at 4 °C. The proteins were incubated with the secondary antibody conjugated with horseradish peroxidase (HRP) and detected by chemiluminescence (Pierce, Rockford, IL, USA). Primary antibodies against TC2N (Cat. #: HPA027549) was purchased from Sigma-Aldrich. Flag (Cat. #: 80010-1-RR and 66008-4-Ig), FASN (Cat. #: 10624-2-AP and 66591-1-Ig), TRIM21 (Cat. #: 12108-1-AP and 67136-1-Ig), PTEN (Cat. #: 22034-1-AP and 60300-1-Ig), LaminA (Cat. #: 10298-1-AP), α-Tubulin (Cat. #: 11224-1-AP), importinβ (Cat. #: 10077-1-AP and 675971-Ig), NEDD8 (Cat. #: 16777-1-AP), NANOG (Cat. #: 14295-1-A), SOX2 (Cat. #: 11064-1-AP) and Ubiquitin (Cat. #: 10201-2-AP) were purchased from Proteintech Group (Wuhan, China). IPO5 (Cat. #: ab187175) was purchased from Abcam (Cambridge, MA, USA). Phospho-Tyrosine (Cat. #: 9411) was purchased from Cell Signaling Technology. Secondary antibodies (Cat. #: A0216 and A0208) were obtained from Beyotime.

### Co-immunoprecipitation (Co-IP) assay

Immunoprecipitation assays were performed using a protein A/G agarose bead (Beyotime, Shanghai, China) according to the manufacturer’s protocol. Briefly, total cell extracts were incubated with the indicated primary antibody and protein A/G agarose beads for 16 h at 4 °C. Next day, the immunoprecipitants were washed at least three times in SDS lysis buffer (Beyotime, Shanghai, China) and further being resolved by SDS-PAGE for WB analyses.

### Transwell assays for cell migration and invasion

Migration and invasion abilities of M231, MCF7 and SKBR3 cells with different TC2N expression were detected using transwell assays in 24-well plates (8 μm, Corning, Acton, MA, USA) with or without Matrigel (BD Bioscience, San Jose, CA, USA). The stably transfected M231, MCF7 and SKBR3 cells were suspended in serum-free medium at 3 × 10^5^ cells/ml, and were seeded in the upper well of the chamber at 0.75 × 10^4^, 3 × 10^4^ and 1 × 10^4^ cells/well, respectively. The media supplemented with 10% FBS were in the lower well of the chamber. The migrated to the lower chamber M231, MCF7 and SKBR3 cells were stained with 0.1% crystal violet, and counted under the microscope after cultured 12 h for migration and invasion. The assays were performed in triplicate for three times.

### Sphere-formation assay

For the sphere-forming assay, single-cell suspensions were plated on 6-well ultralow attachment plates at 1 × 10^4^ cells per well in DMEM/F12 (Gibco) supplemented with B27, epidermal growth factor and basic fibroblast growth factor (R&D Systems). After 8 days of incubation, spheres were counted under an inverted microscope at high magnification. The assays were independently repeated at least three times.

### Flow cytometric analysis

For analysis of CD133 expression, cell-spheres were incubated with fluorescein isothiocyanate (APC)-conjugated anti-human CD133 antibody (eBioscience) according to the manufacturer’s protocol. After incubation for 1 h at 4 °C, the labeled cells were washed thrice with cold PBS and further analyzed using a FACS Calibur flow cytometer (BD Biosciences).

For analysis of CD44^+^CD24^−^ subset, cell-spheres were labeled with anti-CD44 and anti-CD24 antibodies and then incubate with anti-rabbit APC antibody and anti-mouse PE antibody (Bioss, China). CD44^+^CD24^−^ cell populations were gated and sorted out respectively by FACS Calibur flow cytometer (BD Biosciences).

For analysis of ALDH^+^ subpopulation, the ALDEFLUOR assay was performed according to manufacturer’s (STEMCELL Technologies) guidelines. Briefly, a single-cell suspension of BC cells was suspended in ALDEFLUOR buffer containing ALDH subtract and incubated at 37 °C for 30 min. A fraction of cells was incubated under identical condition in the presence of the ALDH inhibitor, diethylamino benzaldehyde (DEAB) to determine the background fluorescence. After the incubation, the cells were wash twice with wash buffer to remove excess ALDH substrate and inhibitors. Thereafter, ALDH^+^ subset was further analyzed using a FACS Calibur flow cytometer (BD Biosciences) according to the instrument’s manual.

### Soft agar clonogenicity assay

Single-cell suspensions were plated on 6-well plates at 200 cells per well in 1 ml of growth media containing 1.2% low-melt agarose and layered onto a 1 ml bed of growth media containing 0.7% low-melt agarose. Cells were fed daily with 200 μl of growth media. After 10 days of incubation, cell colony was stained with 0.05% crystal violet and counted under an inverted microscope.

### Statistical analysis

All replicates displayed in this paper are biological replicates; technical replicates (usually three) were performed and used to generate the means for each biological replicate. The sample sizes and number of replicates were indicated in the figure legends. Statistical analyses were performed using SPSS 19.0 software (SPSS, Inc., Chicago, IL, USA) and GraphPad Prism 9 software (La Jolla, CA, USA). All data were presented as means ± standard error of the mean (SEM). The differences between groups were assessed by Chi-square test, Student’s t-test (only two groups) or One-way ANOVA (three or four groups). The correlations were analyzed using Pearson and Spearman correlation test. Survival analyses were calculated by log-rank test Kaplan–Meier and multivariate COX-regression analyses. The P-values of < 0.05 was considered to be statistically significant.

## Results

### The expression of TC2N is associated with metastatic characteristic and differentiating degree of BC patients

**Fig. 1:**
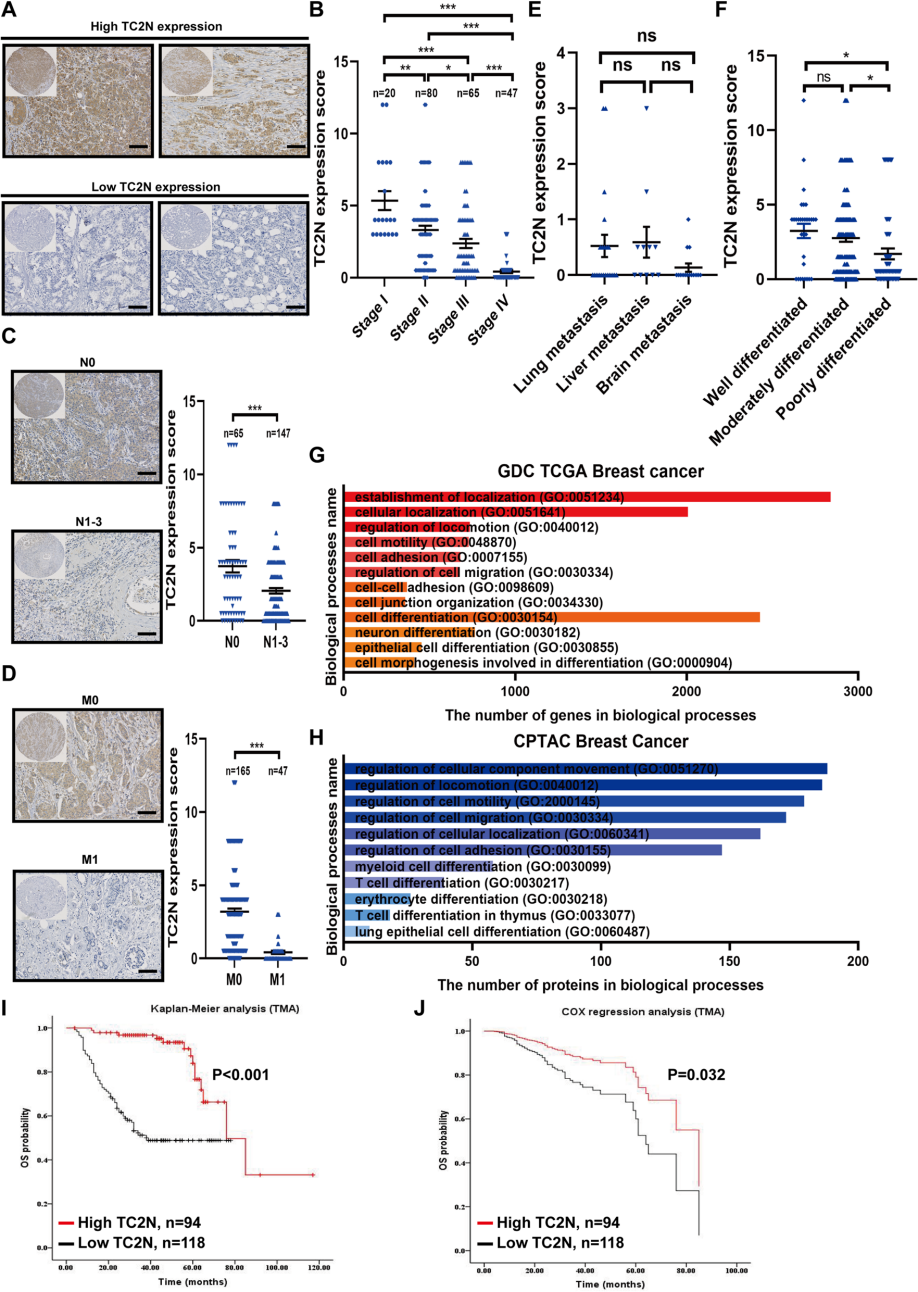
TC2N expression is related to tumor metastasis and differentiation status of BC patients. **A** The protein expression of TC2N was detected by IHC staining in BC tissues. High TC2N expression group contains the ≥ 3 score patients. Low TC2N expression group contains the < 3 score patients. Scale bars represent 20 μm. **B** TC2N expression was significantly decreased in advanced-stage BC patients than that in early-stage BC patients. The P value was measured with Student’s t-tests. *P < 0.05; **P < 0.01; ***P < 0.001. **C** TC2N expression was obviously decreased in BC patients with lymph node metastasis (N_1–3_) than that without lymph node metastasis (N_0_). The P value was measured with Student’s t-tests. ***P < 0.001. **D** TC2N expression was significantly decreased in BC patients with distant-metastasis (M_1_) than that without metastasis (M_0_). The P value was measured with Student’s t-tests. ***P < 0.001. Scale bars represent 20 μm. **E** The expression of TC2N in different metastatic sites. The P value was measured with Student’s t-tests. ns, no significance. **F** TC2N expression in BC tissues at different differentiating degree. The P value was measured with Student’s t-tests. ns, no significance, *P < 0.05. **G** TCGA BC database identified the association between TC2N expression and metastasis and differentiation-related processes. **H** CPTAC BC protein dataset identified the association between TC2N expression and metastasis and differentiation-related processes. **I** Kaplan–Meier analysis of the correlation between TC2N expression and overall survival time in 212 BC patients. **J** Cox-regression analysis of the correlation between TC2N expression and overall survival time in 212 BC patients

The small samples of BC limited our analysis of the clinicopathologic significance of TC2N in previous study. Thus, we executed a large sample size of tissue microarray (TMA) that contains 212 BC specimens to further evaluate the clinical value of TC2N expression. The expression of TC2N were classified into two groups: TC2N high expression (scores 3 ≤ and ≤ 12) and TC2N low expression (scores <3) (Fig. 1A). Through further statistical analyzing the association of TC2N protein expression with clinicopathological characteristic, we found TC2N expression is related to clinical stage, differentiating degree, tumor invasion depth, molecular subtype, especially lymph node metastasis and distant metastasis (Table 1). The percentage of TC2N high expression samples was high in clinical early stages (I + II), reaching 67% (67/100), compared with 24.11% (27/112) of advanced stages (IV + III) (Table 1). The TC2N expression decreased in an order from clinical stage I, II, III to IV groups (Fig. 1B). Moreover, compared with patients without lymph nodal metastasis (N_0_), TC2N expression was obviously reduced in the patients with lymph nodal metastasis (N_1-3_) (Fig. 1C). Next, we investigated the percentage of TC2N high expression in patients with or without distant metastasis and found that the percentage of TC2N high-expression samples in distant metastasis group (M_1_) was significantly lower than that in non-metastasis group (M_0_), 6.38% (3/47) vs. 55.15% (91/165), respectively (Table 1). TC2N expression was much low in the group of metastases compared to that of non-metastasis (Fig. 1D). Especially, brain metastasis exhibited lower mean expression level of TC2N than other metastatic lesions, though the differences between each site were not statistically significant (Fig. 1E). Besides, we also observed that with an increasing differentiating degree of BC, the expression of TC2N increased (Table 1 and Fig. 1F), indicating an underlying relationship between TC2N expression and cell differentiation. To further identify the above observation, the genes or proteins with same expression pattern of TC2N were screened using R project (Additional file 2: Data S1) base on two public databases, The Cancer Genome Atlas (TCGA) and Clinical Proteomic Tumor Analysis Consortium (CPTAC) respectively. Further Gene ontology (GO) enrichment analysis was performed based on these co-expressed genes and proteins, and showed that co-expressed molecules of TC2N were correlated with metastasis and cell differentiation (Fig. 1G, H). Then, these results were supported by 26 large independent sample GEO datasets (n ≥ 100) (Additional file 2: Data S2 and Additional file 7: Fig. S1A).

**Table 1:** Association of TC2N expression with BC clinicopathological characteristics

Variable	Category	Relative TC2N expression		P
		High (n = 94)	Low (n = 118)	
Age (years)	≤ 48	51	55	0.269
	> 48	43	63	
Clinical stage (AJCC)	I	**20**	**0**	**< 0.001**
	II	**47**	**33**	
	III	**24**	**41**	
	IV	**3**	**44**	
Differentiating degree	High	**21**	**10**	**< 0.001**
	Moderate	**61**	**69**	
	Low	**12**	**39**	
Depth of tumor invasion	T_1_	**31**	**13**	**< 0.001**
	T_2_	**38**	**41**	
	T_3_	**24**	**51**	
	T_4_	**1**	**13**	
Lymph node metastasis	N_0_	**38**	**27**	**0.006**
	N_1-3_	**56**	**91**	
Distant metastasis	M_0_	**91**	**77**	**< 0.001**
	M_1_	**3**	**41**	
Primary site	Left breast	60	77	0.829
	Right breast	34	41	
Disease type	Ductal BC	81	100	0.771
	Lobular BC	13	18	
Molecular subtype	Luminal A	**26**	**33**	**< 0.001**
	Luminal B	**31**	**23**	
	HER2 +	**21**	**19**	
	Basal-like	**16**	**43**	

Bolded values indicate statistical significance, P < 0.05

To analyze its prognostic significance, Kaplan–Meier analysis and COX regression analysis were performed based on TMA and revealed that TC2N indicated good prognosis in human BC (Fig. 1I, J). Thereafter, the similar results were obtained in two public databases, Kaplan–Meier Plotter and GSE25307 (Additional file 7: Fig. S1B, C). Taken together, these results suggest a role for TC2N in inhibiting the progression of BC.

### TC2N inhibits the migration and invasion phenotype of BC cells in vitro and suppresses metastasis in vivo

**Fig. 2:**
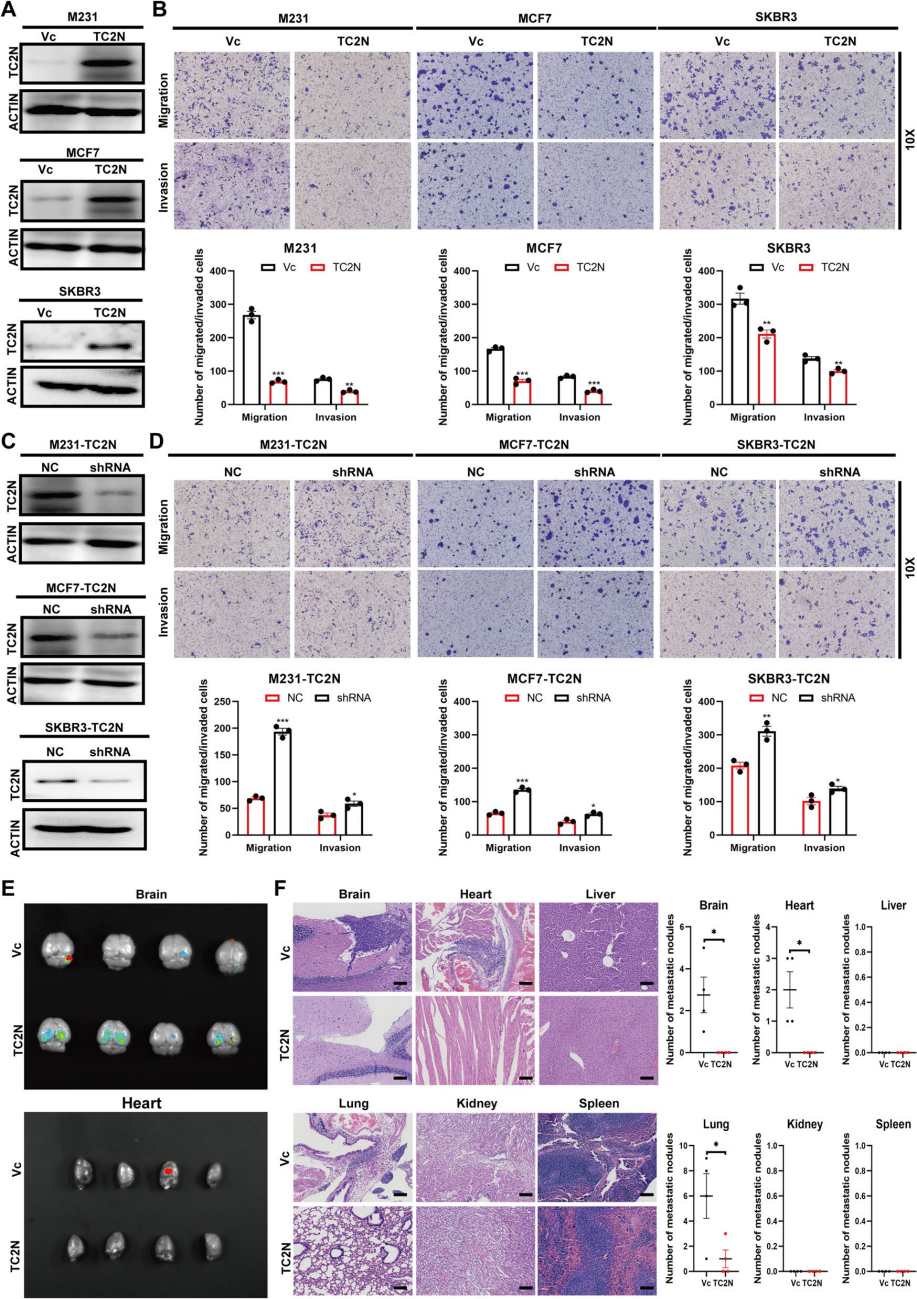
TC2N inhibits metastatic phenotype of BC cells in vitro and in vivo. **A** Overexpression of TC2N in M231, MCF7 and SKBR3 cells were examined by WB. **B** Effects of TC2N overexpression on migration and invasion of M231, MCF7 and SKBR3 cells were detected by transwell assays. Mean ± SEM (n = 3). The P value was measured with Student’s t-tests. **P < 0.01, ***P < 0.001. **C** Silencing of TC2N in M231-TC2N, MCF7-TC2N and SKBR3-TC2N stable transfectants were examined by WB. **D** Effects of TC2N knockdown on migration and invasion of M231-TC2N, MCF7-TC2N and SKBR3-TC2N cells were detected by transwell assays. Mean ± SEM (n = 3). The P value was measured with Student’s t-tests. *P < 0.05, **P < 0.01, ***P < 0.001. **E** Bioluminescent images of brain and heart tissues from female nude mice 50 days post intracardiac injection of SKBR3 cell line with TC2N ectopic expression. **F** Tissues were exfoliated from mice (n = 4) that injected with stable transfected SKBR3 cells. Organs invaded by BC cells were evaluated by H&E staining (Left). Scale bar represent 100 μm. The number of metastatic nodules in each organ were showed (Right). The P value was measured with Student’s t-tests. *P < 0.05

Considering the TC2N expression is related to metastatic status of patients, we asked the question if TC2N is important for the anti-metastasis of BC. After the establishment of three TC2N-overexpressing stable BC cell lines MDA-MB-231 (M231)-TC2N, MCF7-TC2N and SKBR3-TC2N (Fig. 2A), the migrative and invasive activity of these cells were determined by Transwell assays. As expected, upregulation of TC2N markedly restricted cell migration and invasion of BC cells (Fig. 2B). To further confirm the role of TC2N in BC, the effect of TC2N silencing on cell migration and invasion in three stable transfectants (M231-TC2N, MCF7-TC2N and SKBR3-TC2N) with TC2N shRNA were investigated (Fig. 2C). In parallel, knockdown of TC2N recovered the migrative and invasive potential in these cells (Fig. 2D). These data suggest that TC2N sharply suppresses cell migration and invasion of BC.

Given the TC2N expression was downregulated in patients with metastasis, especially in brain metastasis loci, we further created a in vivo metastasis model by injection of highly metastatic SKBR3 cells with stable overexpression of TC2N-tagged EGFP or vector control into the left cardiac ventricle of nude mice, respectively, to examine the impact of TC2N on metastasis. Strikingly, the fluorescence signal of metastatic foci of the brain and heart were more easily detected in vector control group than those in TC2N group (Fig. 2E). For a more direct assessment of metastatic potential, we performed pathologic examination to observe metastatic nodules in multiple organs, including brain, heart, liver, lung, kidney and spleen of mice by HE staining. We found that cells with TC2N enforced expression produced less and smaller metastatic foci in the brain, heart and lung compared to control cells (Fig. 2F). However, no obvious lesion was observed in the liver, kidney and spleen (Fig. 2F).

### TC2N overexpression attenuates stemness of BC cells in vitro and in vivo

**Fig. 3:**
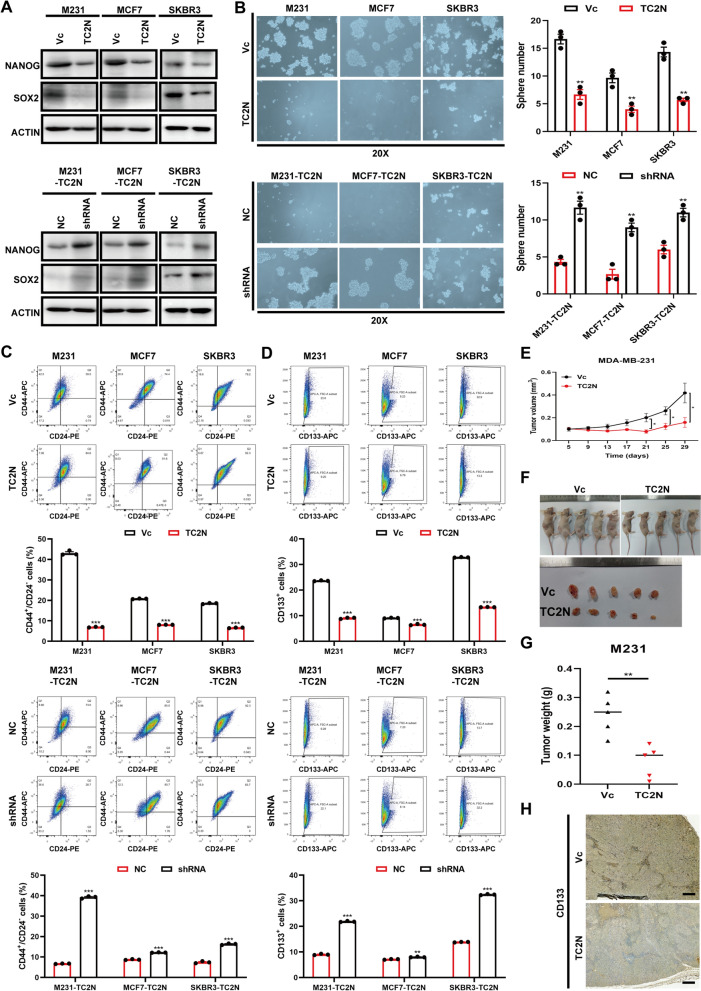
TC2N interferes with the CSC-like characteristics of BC cells. **A** WB analysis of NANOG and SOX2 in stable M231, MCF7 and SKBR3 cells. **B** Sphere formation ability of BC cells with ectopic expression of TC2N. Mean ± SEM (n = 3). The P value was measured with Student’s t-tests. **P < 0.01. **C** Fractions of CD44^+^CD24^−^ BC cell-spheres with TC2N ectopic expression were determined by flow cytometry. Mean ± SEM (n = 3). The P value was measured with Student’s t-tests. ***P < 0.001. **D** Fractions of ADLH^+^ BC cells with TC2N ectopic expression were determined by flow cytometry. Mean ± SEM (n = 3). The P value was measured with Student’s t-tests. **P < 0.01, ***P < 0.001. **E** The tumor growth curves of M231 spheres (n = 5). *P < 0.05. **F** The image of nude mice subcutaneously injected with M231 spheres. **G** Tumor weight from the Vc and TC2N group. **P < 0.01. **H** IHC evaluation of CD133 in xenograft tumor. Scale bar represent 100 μm

Analogically, above clinic correlation about TC2N and differentiation spur us on to explore the function of TC2N in breast cancer stem cells (BCSCs). M231-TC2N, MCF7-TC2N and SKBR3-TC2N cells were cultured in serum-free suspension medium to induce spheroids. After isolation and purification of a subpopulation of spheroid‐forming cells (Additional file 8: Fig. S2A), the TC2N expression was examined. We observed that TC2N protein levels were decreased in BC-spheres (Sp) compared with monolayer cells (Ad) (Additional file 8: Fig. S2A), hinting a potential role of TC2N in BCSCs. To further explore this assumption, the expression of NANOG and SOX2, two key transcriptional factors driving the stemness properties [25] were detected in TC2N stable overexpression or knockdown BC cells. Consistently, overexpressing TC2N significantly reduced the expression of NANOG and SOX2, which could be rescued by silencing of TC2N (Fig. 3A). We also observed that overexpression of TC2N greatly reduced the number and size of spheres, whereas knockdown of TC2N formed much larger and significantly more spheres (Fig. 3B). Meanwhile, the CD44^+^CD24^−^ subsets of BC-spheres and another CSC marker CD133 that could serve as a stem cell marker for BC [28] were monitored by flow cytometry analysis. The results showed that the percent of CD44^+^CD24^−^ and CD133-positive cell spheres decreased with TC2N overexpression, and increased with TC2N interference (Fig. 3C and D). Subsequently, ALDH^+^ subpopulations of BC cells which have been reported to enrich BCSCs were significantly reduced or increased upon TC2N overexpression or silencing (Additional file 8: Fig. S2B and C). Furthermore, soft agar colony formation assay was performed to further assess self‐renewal of BCSCs. Unsurprisingly, cell spheres with high TC2N expression formed less clone than that with low TC2N expression (Additional file 8: Fig. S2D and E). In addition, when compared with the percentage of EdU^+^ spheres among blank spheres, the EdU^+^ percentages were significantly lower among BC spheres with TC2N overexpression (Additional file 8: Fig. S2F). These observations indicate that TC2N impedes the self‐renewal ability and clonogenic activity of BC cells in vitro.

To gain insights into the importance of TC2N in suppressing the stem cell-like properties of BC cells in vivo, BC cell-spheres were subcutaneous injected into nude mice. The tumor growth, size and weight of TC2N-overexpressed BC-spheres were inhibited compared to control group (Fig. 3E–G). IHC analysis displayed a decreased expression of CD133 in tumor tissues after TC2N overexpression (Fig. 3H). These data represented the inhibitory function of TC2N in the CSC-like phenotype.

### Upregulation of TC2N suppresses fatty acid synthesis in BC

**Fig. 4:**
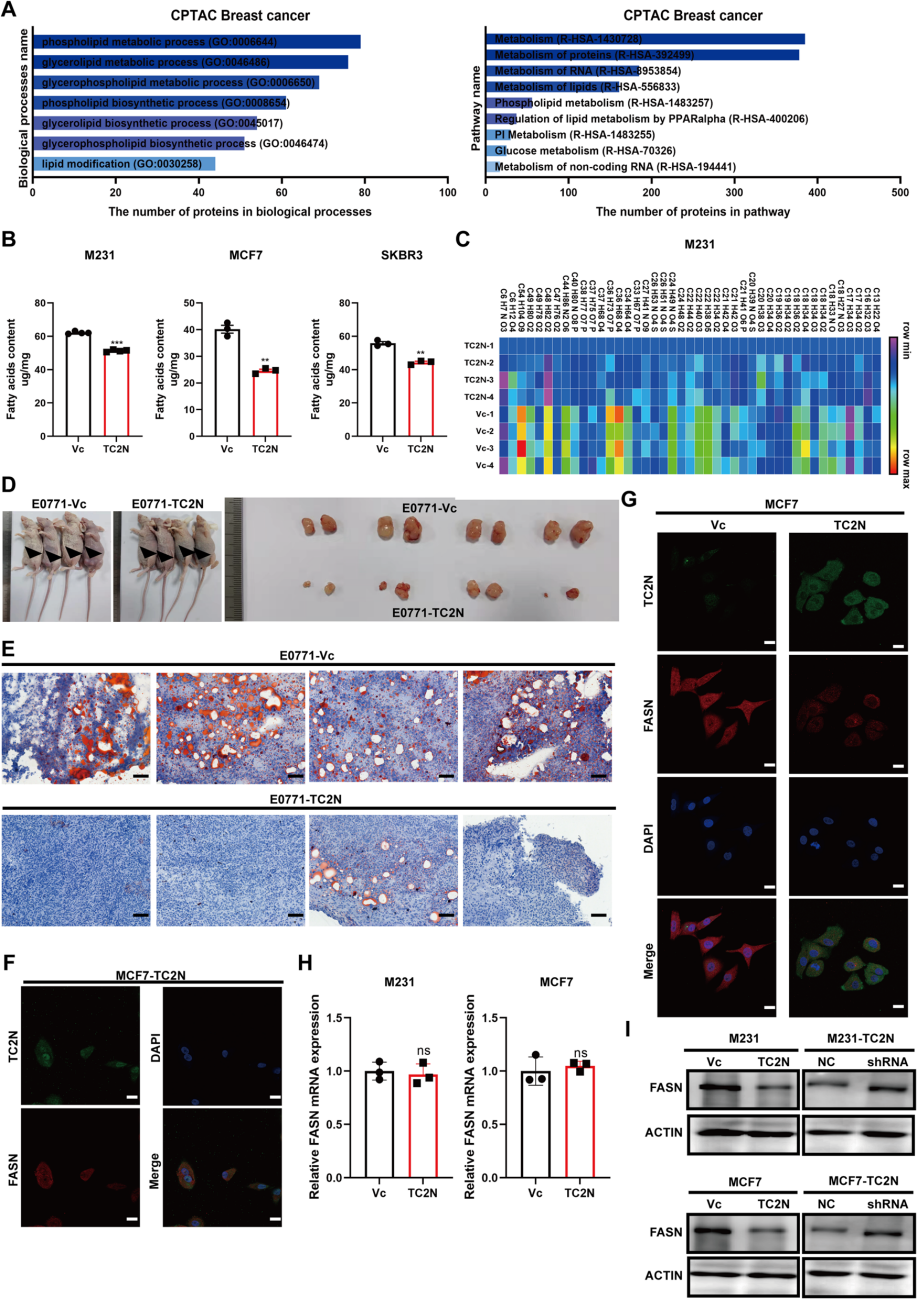
TC2N decreases the fatty acid content in BC. **A** CPTAC BC protein dataset identified the association between TC2N expression and lipid metabolism. **B** Effects of TC2N overexpression on total free fatty acids levels of M231, MCF7 and SKBR3 cells. The P value was measured with Student’s t-tests. **P < 0.01, ***P < 0.001. **C** Lipid metabolism was detected by LC–MS/MS in stable transfected M231 cells. Heatmap was performed and the significant differences were analyzed using Student’s t-test, n = 4. **D** The growth of E0771 cells stably expressing control lentivirus or TC2N overexpression lentivirus in nude mice. Block arrows indicate the tumor masses. **E** The fatty deposit of E0771 tumor masses were detected by oil red O staining. Scale bar represent 100 μm. **F** Immunofluorescence analysis of the localization of TC2N and FASN in MCF7-TC2N cells. DAPI serves as a nuclear counterstain. Scale bars represent 25 μm. **G** Immunofluorescence revealed that TC2N decreases the protein levels of FASN in MCF7 cells. Scale bars represent 25 μm. **H** qRT-PCR revealed that TC2N do not change the mRNA expression of FASN in M231 and MCF7 cells. The P value was measured with Student’s t-tests. ns, no significance. **I** WB revealed that TC2N decreases the protein expression of FASN in M231 and MCF7 cells

To investigate the underlying molecular mechanism of TC2N in BC, Gene set enrichment analysis (GSEA) was performed using RNA-Seq gene expression data from TCGA database and aforementioned 26 large sample GEO datasets. The results of GSEA reveal that there are significant differences (Nominal P value < 0.05) in enrichment of biological processes between high-TC2N-expression tumors and low-TC2N-expression tumors. Interestingly, we found that TC2N expression significantly correlated with the “FATTY_ACID_METABOLISM” or “LIPID _METABOLISM” process in both TCGA and 26 public datasets (Additional file 4: Data S3 and Additional file 9: Fig. S3A-D). These results were further confirmed by performing GO and Reactome pathway enrichment analyses in the protein level (Fig. 4A). Knowing the importance of fatty acid in tumor progression, we further focused on whether TC2N can regulate fatty acid metabolism in BC. Fatty acid analysis revealed that the total fatty acids level of BC cells decreased significantly in the TC2N-overexpressed BC cells compared to those in the control groups (Fig. 4B). Furthermore, extended metabolomic analysis focusing on the consists of fatty acids revealed that TC2N overexpression leads to limit a variety of fatty acid synthesis (Fig. 4C, Additional file 5: Data S4). To test TC2N inhibitory function on fatty acid synthesis in vivo, we injected E0771 cells into the mammary fat pad of nude mice to produce tumor models of breast cancer in situ and further observed lipid deposition by oil red O staining. Consistently, TC2N overexpression impeded tumor growth (Fig. 4D), accompanying decreased fat content in tumor tissues (Fig. 4E). Owing to our previous work showing that a critical fatty acid synthase, FASN, is a potential interactor of TC2N in our pervious study [18], we first sought to refine this observation. For clarity, the subcellular localization of TC2N and FASN were detected by fluorescent microscopy. Remarkably, we observed that TC2N was co-localized with FASN in both the cytoplasm and nucleus of stable transfected BC cells (Fig. 4F). Additionally, we noticed that the expression of FASN was lower in TC2N overexpressing BC cells than in control BC cells (Fig. 4G). Hence, we speculate that TC2N may attenuate fatty acid synthesis via regulation of FASN expression. Through detecting the expression change of FASN after TC2N overexpression or knock-down, we found TC2N significantly enhance FASN protein levels (Fig. 4I), while the mRNA level of FASN was unaffected (Fig. 4H).

### TC2N dramatically promotes FASN protein degradation by interfering with PTEN binding to FASN upon high glucose condition

**Fig. 5:**
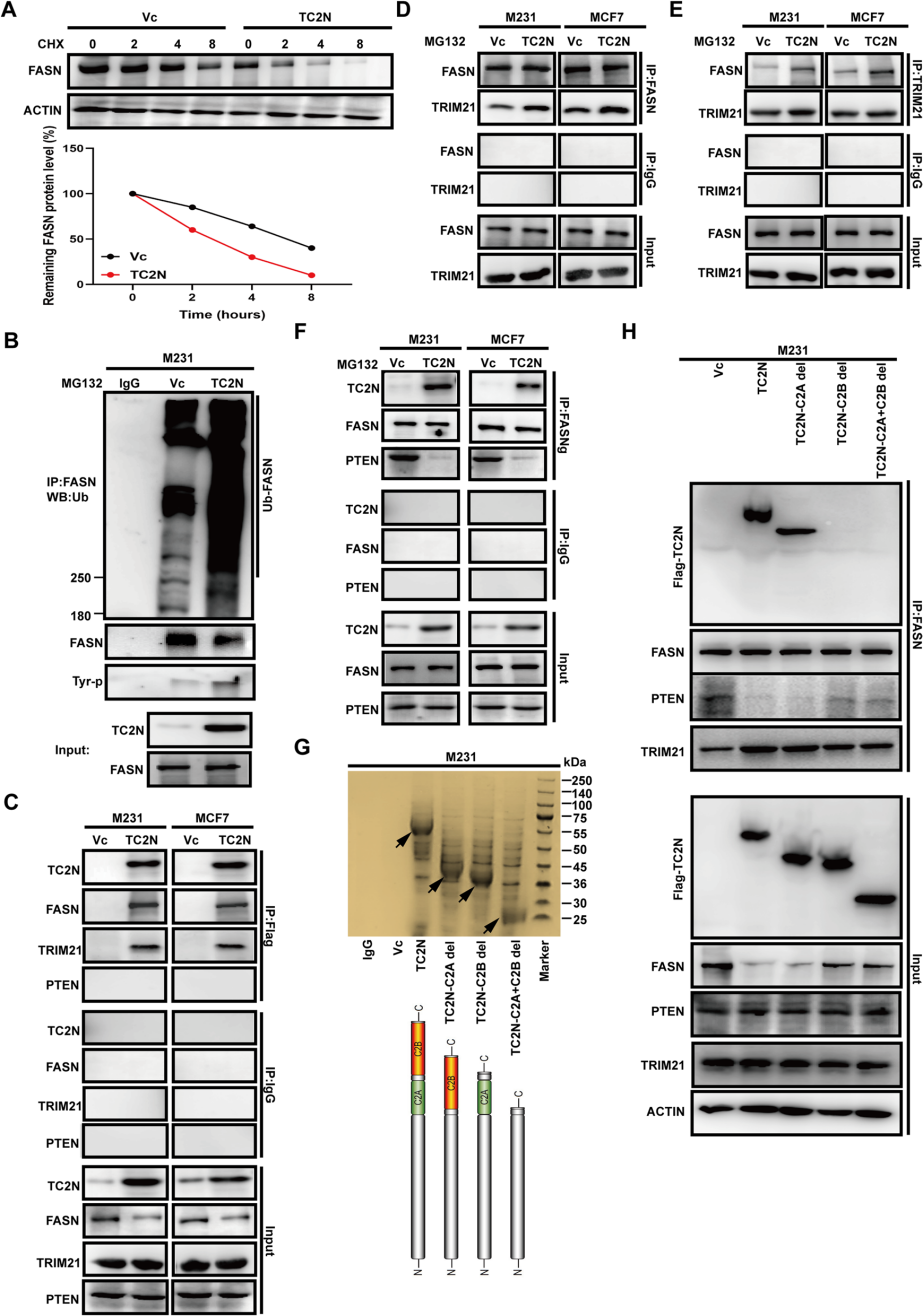
TC2N promotes the degradation of FASN via blocking the interaction between PTEN and FASN. **A** The extracts from M231 cells with control lentivirus or TC2N overexpression lentivirus treated with 20 µM cycloheximide (CHX) for the indicated times were subjected to WB (Upper). Relative FASN protein levels were quantified by ImageJ software (Lower). **B** M231 cells with control lentivirus or TC2N overexpression lentivirus were transfected with 2 µg of ubiquitin-expressing plasmids. At 24 h after the transfection, cells were treated with 20 µM MG132 for 24 h. Cell extracts were subjected to IP with anti-FASN antibody and further analyzed by WB with anti-Ubiquitin and anti- Tyrosine antibody. Normal IgG was used as a negative control. Whole-cell lysates were used as a positive control (Input). **C** The extracts from BC cells were subjected to IP with anti-Flag antibody and further analyzed by WB with TC2N, FASN, TRIM21 and PTEN antibodies. Normal IgG was used as a negative control. Whole-cell lysates were used as a positive control (Input). **D** and **E** BC cells were treated with 20 µM MG132 for 24 h, and then cell extracts were subjected to IP with anti-FASN or anti-TRIM21 antibodies and further analyzed by WB with indicated antibodies. Normal IgG was used as a negative control. Whole-cell lysates were used as a positive control (Input). **F** The cell extracts from BC cells were subjected to IP with anti-FASN antibody and further analyzed by WB with indicated antibodies. Normal IgG was used as a negative control. Whole-cell lysates were used as a positive control (Input). **G** The extracts from M231 cells stably expressing FLAG-Vc or FLAG-TC2N (full-length and truncation) were subjected to IP with anti-Flag antibody. Elutes were resolved using SDS-PAGE and silver-stained. Block arrows indicate the bands of TC2N. **H** The extracts from M231 cells stably expressing FLAG-Vc or FLAG-TC2N (full-length and truncation) were subjected to IP with anti-FASN antibody and further analyzed by WB with indicated antibodies. Normal IgG was used as a negative control. Whole-cell lysates were used as a positive control (Input)

FASN is essential for de novo fatty acid synthesis to support metastatic progression and stemness of human cancers [29, 30]. Thus, we conclude that TC2N inhibits invasion and metastasis by block of the fatty acid synthase. Here, the expression changes of FASN only occurred at the protein level and raised the possibility that TC2N affects its protein stability. As shown in Fig. 5A, the half-life of FASN protein was shortened in BC cells with TC2N overexpression. However, the mechanism about how TC2N induce FASN degradation is still unknow. TRIM21, a known E3 ubiquitin ligase, can repress lipid metabolism via inducing ubiquitylation and degradation of FASN [31, 32]. Moreover, new research declared that high glucose can switch PTEN from a tumor suppressor to a tumor promoter by triggering its neddylation, and then this neddylated PTEN move to the nucleus and decrease the association of FASN with TRIM21 via dephosphorylating FASN in BC [33]. Considering the proteomic data from our previous studies indicated that TRIM21 but not PTEN as a underlying interactor of TC2N [18], we further hypothesized that TC2N may involve in these events. Indeed, TC2N enhanced tyrosine phosphorylation and ubiquitination of FASN (Fig. 5B). Furthermore, IP analysis verified that under high glucose condition, TC2N interacted with FASN and TRIM21, but there was no interaction between TC2N and PTEN in our TC2N-overexpressing BC cell model (Fig. 5C). Subsequently, by using FASN or TRIM21 antibodies, increased levels of endogenous TRIM21 protein were detected in FASN precipitates upon TC2N overexpression and vice versa (detect FASN in TRIM21 precipitates) (Fig. 5D, E). To better illustrate the regulatory mechanism, further co-IP assays were performed in BC stable cells. The results indicated that when TC2N was overexpressed, FASN favored the interaction with TC2N but not with PTEN in the presence of high levels of glucose (Fig. 5F). These data suggest that TC2N can strengthen the interaction of TRIM21 with FASN by competing with the binding of PTEN to FASN in nucleus. Next, to confirm the key domain of TC2N is responsible for its interaction with FASN, co-IP assays were performed in BC cells infected with TC2N full-length, TC2N-C2A del (lack C2A domain of TC2N, C2A-del), TC2N-C2B del (lack C2B domain of TC2N, C2B-del) and TC2N-C2A + C2B del (lack C2A and C2B domain of TC2N, C2A + C2B-del) lentivirus, respectively (Fig. 5G). As shown in Fig. 5H, full-length TC2N and C2A-del but not C2B-del and C2A + C2B-del interacted with FASN. This result demonstrates that the C2B domain of TC2N is necessary for interaction of FASN.

Remarkably, we noticed that only part of the protein level of FASN was rescued by C2B domain deletion (Fig. 5H). In addition, when compared with full-length TC2N and C2A-del groups, the interaction between FASN and PTEN was partly increased, and consequently, the interaction between FASN and TRIM21 was partly decreased within C2B-del and C2A + C2B-del groups (Fig. 5H), suggesting a role of TC2N in regulation of PTEN neddylation. As the C2B domain is required for transporting TC2N into the nucleus [34], we inferred that cytoplasmic TC2N also contribute to the degradation of FASN by orchestrating neddylated PTEN.

### TC2N inhibits PTEN neddylation through preventing PTEN nuclear import by interacting with import proteins

**Fig. 6:**
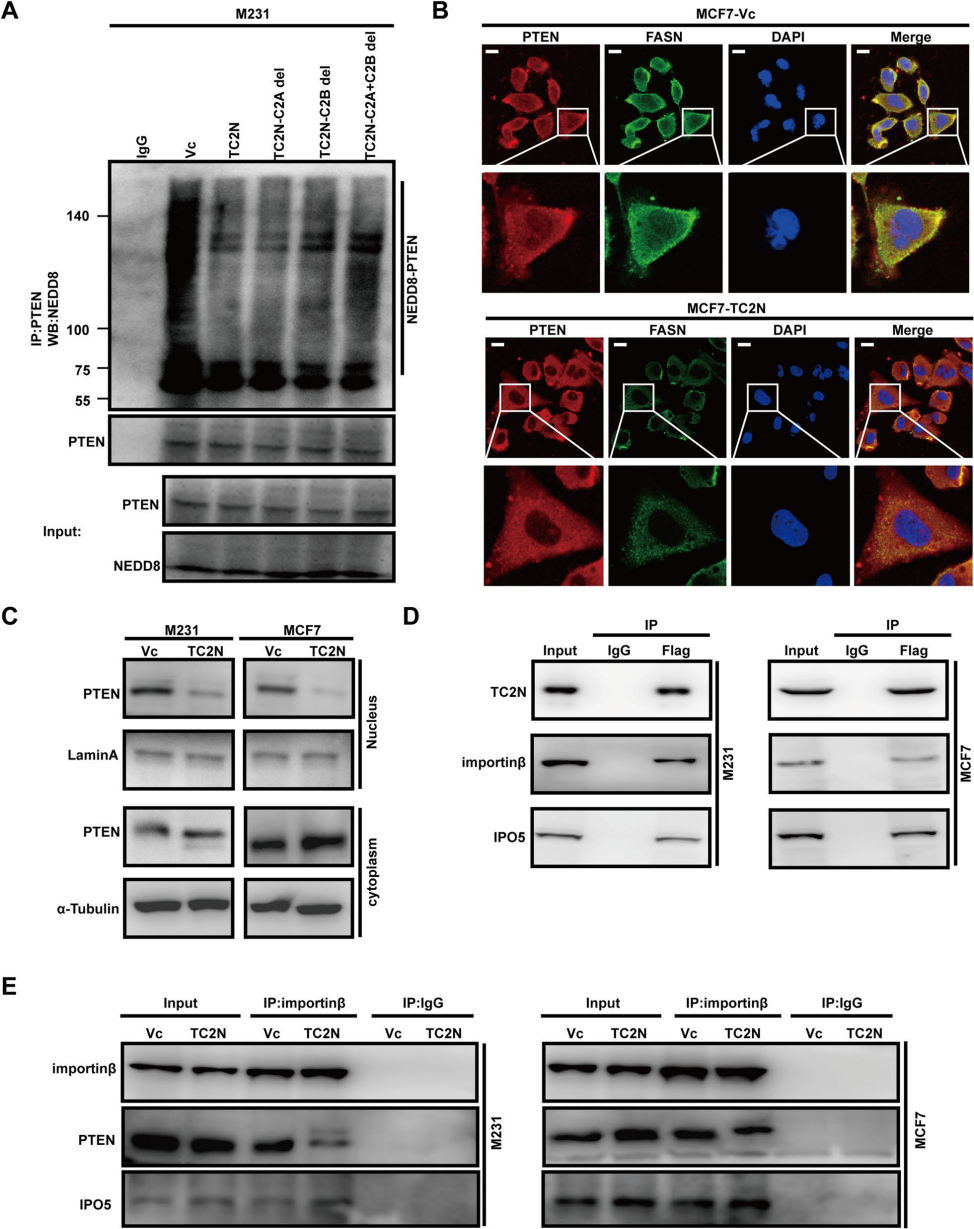
TC2N decreases the levels of PTEN neddylation by preventing the nuclear import of PTEN. **A** M231 cell extracts were subjected to IP with anti-PTEN antibody and further analyzed by WB with anti-NEDD8 antibody. Normal IgG was used as a negative control. Whole-cell lysates were used as a positive control (Input). **B** Immunofluorescence revealed that TC2N decreases the nuclear localization of PTEN in MCF7 cells. Scale bars represent 25 μm. **C** PTEN protein level in nucleus and cytoplasm were analyzed by WB. **D** The extracts from BC cells were subjected to IP with anti-Flag antibody and further analyzed by WB with anti-importinβ and anti-IPO5 antibodies. Normal IgG was used as a negative control. Whole-cell lysates were used as a positive control (Input). **E** The cell extracts from BC cells were subjected to IP with importinβ antibody and further analyzed by WB with indicated antibodies. Normal IgG was used as a negative control. Whole-cell lysates were used as a positive control (Input)

To illustrate the effect of TC2N on PTEN neddylation, we monitored neddylated PTEN level in BC cells with overexpression of full-length or truncated TC2N. As shown in Fig. 6A, all full-length and truncated TC2N can markedly decreased PTEN neddylation, and the neddylation levels of PTEN between full-length and truncated TC2N did not show major difference. These results confirm that cytoplasmic rather than nuclear TC2N effectively decreased PTEN neddylation. Considering that nuclear translocation of PTEN is a critical step in forming neddylated PTEN [33], we next sought to examine whether TC2N affect the subcellular localization of PTEN. Immunofluorescence analysis showed that TC2N overexpression attenuates the co-localization of PTEN and FASN in the nucleus (Fig. 6B). Consistently, further cell fractionation analysis confirmed this conclusion. In the absence of TC2N, PTEN was localized to both the cytoplasm and nucleus. Notably, in cells overexpressing TC2N, PTEN was predominantly localized to the cytoplasm (Fig. 6C). These data suggest that TC2N tends to regulates the subcellular localization of PTEN, but the mechanism involved remains completely unknown. The nuclear pore complex (NPC) which includes importin proteins are necessary for nuclear protein import [35]. We noticed that two importin proteins were identified as a potential TC2N-interacting protein in our previous work [18]. Indeed, TC2N was coimmunoprecipitated with importinβ and IPO5 in BC cells (Fig. 6D), and overexpression of TC2N weakened the interaction of importin with PTEN (Fig. 6E), suggesting that TC2N controls the binding of importin to PTEN. These data provide a potential mechanism of how TC2N affects the neddylation and nuclear translocation of PTEN.

### FASN activity is responsible for the role of TC2N in BC

**Fig. 7:**
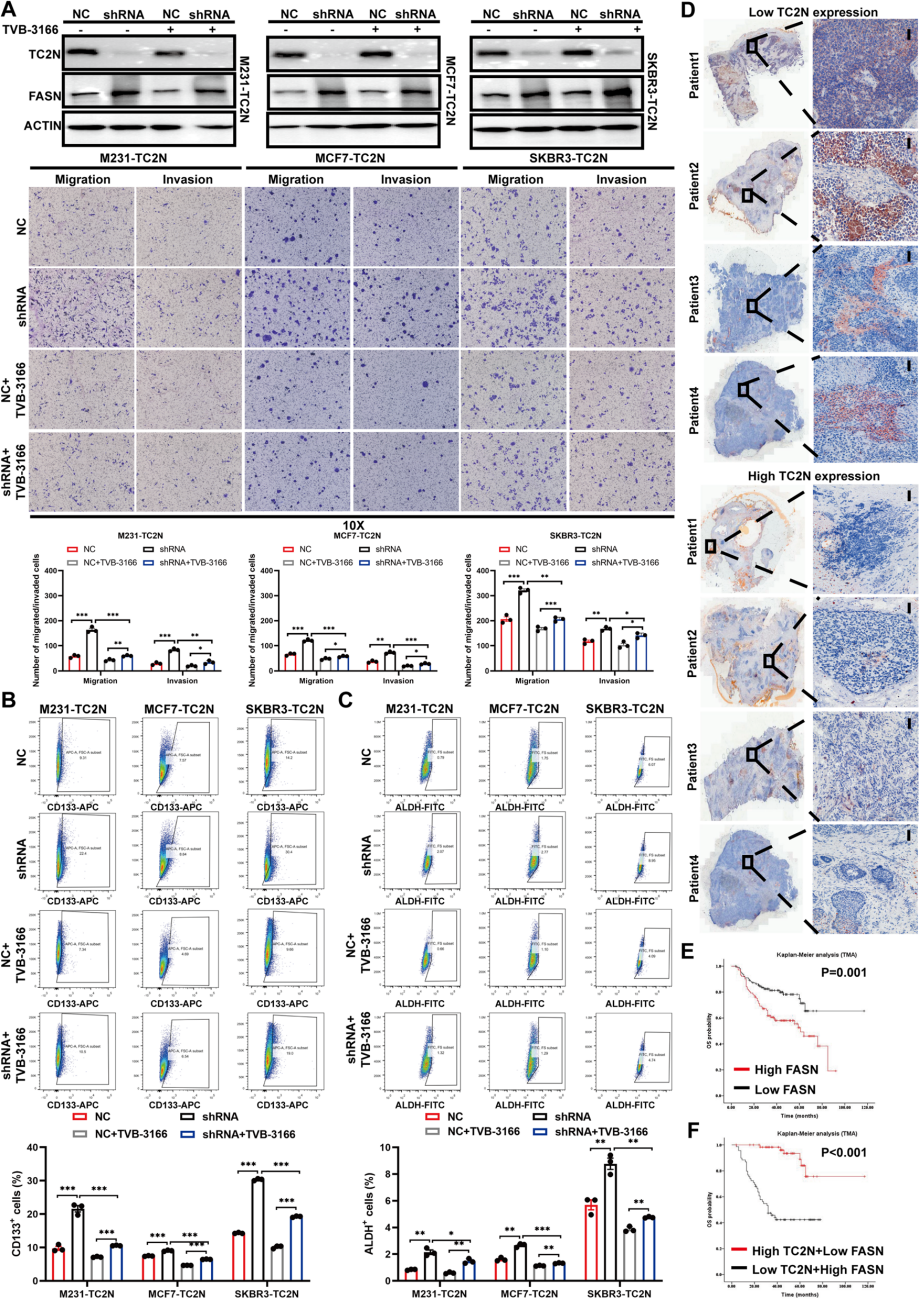
TC2N represses FASN-driven tumor progression and its high expression in BC indicate low fatty deposition and good prognosis. **A** WB analysis of TC2N and FASN expression in stable M231, MCF7 and SKBR3 cells with 0.2 μM TVB-3166 treatment (Upper). Transwell assays were used to examine the effect of FASN ablation on cell migration and invasion of BC stable cells (Under). The P value was measured with Student’s t-tests. *P < 0.05, **P < 0.01, ***P < 0.001. **B** CD133^+^ analysis was used to examine the effect of FASN ablation on stemness of BC stable cell spheres. Mean ± SEM (n = 3). The P value was measured with Student’s t-tests. ***P < 0.001. **C** ALDH^+^ analysis was used to examine the effect of FASN ablation on stemness of BC stable cells. Mean ± SEM (n = 3). The P value was measured with Student’s t-tests. *P < 0.05, **P < 0.01, ***P < 0.001. **D** The fatty deposits were detected by oil red O staining in tumor tissues of BC patients. Scale bar represent 100 μm. **E** and **F** Survivorship curves showed that the overall survival rates of BC patients with high or low levels of FASN and of those with low expression of TC2N and high expression of FASN or high expression of TC2N and low expression of FASN. The high or low levels of TC2N and FASN were defined by their median levels in the BC

FASN is essential for de novo fatty acid synthesis to support metastatic progression and stemness of human cancers [29, 30]. Having determined that TC2N is the upstream regulator of FASN, we conclude that TC2N exerts inhibitory function possibly by block of the fatty acid synthase. As shown in Fig. 7A, a clinical grade FASN inhibitor, TVB-3166, significantly lessened the migrated and invaded BC cells induced by TC2N knockdown. On the other hand, inhibition of FASN attenuated the inductive effects of TC2N silence on stemness (Fig. 7B and C). Collectively, these findings suggest that FASN contribute to the function of TC2N in BC.

**Fig. 8:**
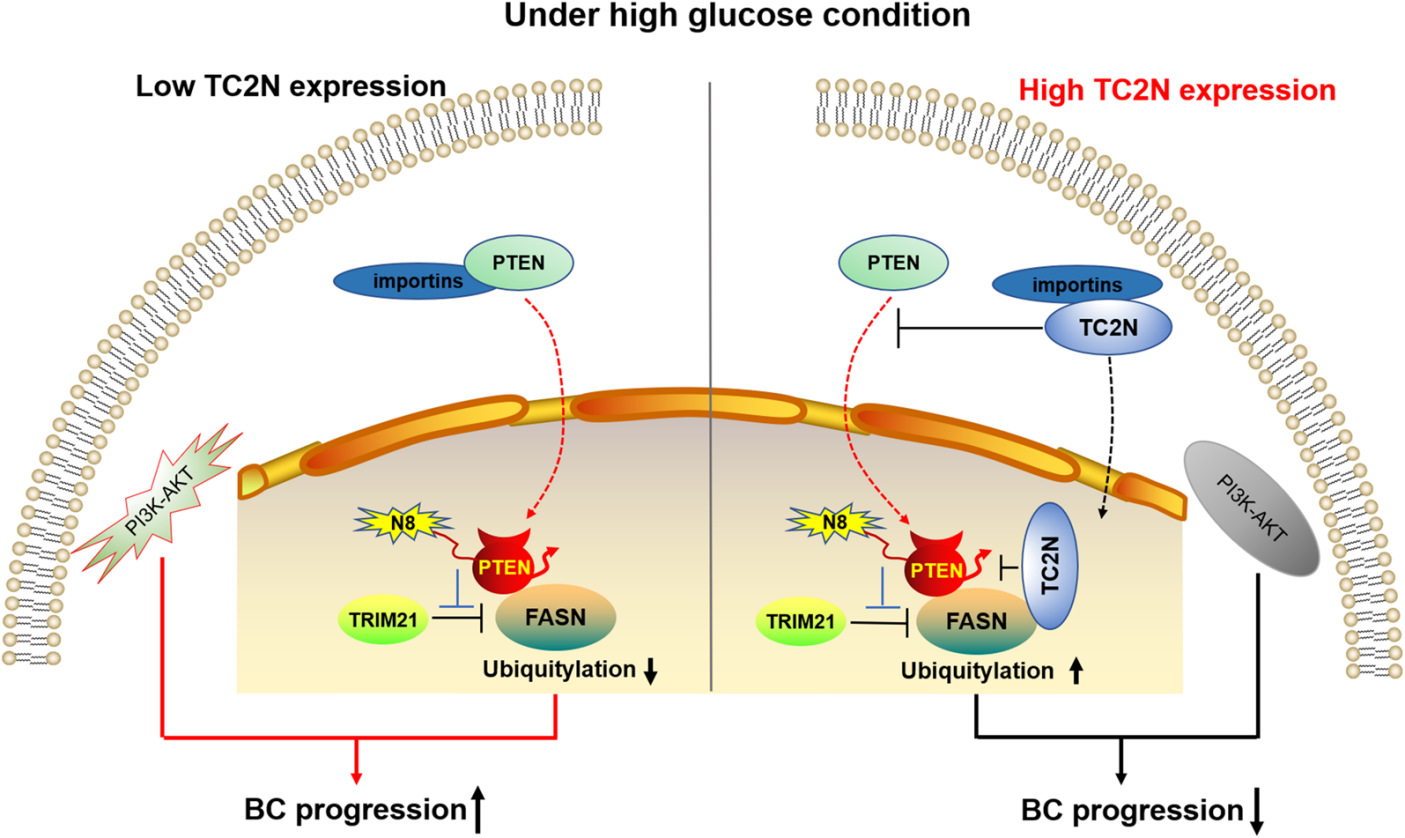
A schematic diagram reveals the regulatory mechanism by which TC2N induces FASN degradation

Clinically, patients with high levels of TC2N showed lower fatty acid content, and, conversely, patients with low levels of TC2N showed higher fatty acid content (Fig. 7D). Furthermore, we assessed the prognostic significance of FASN in BC through Kaplan–Meier survival analysis. BC patients with high FASN protein levels exhibited a poorer trend in the overall survival rates than those with low FASN protein levels (Fig. 7E). Further, these BC patients were divided into the following four groups based on the median levels of TC2N and FASN protein: (1) high TC2N expression and high FASN expression, (2) high TC2N expression and low FASN expression, (3) low TC2N expression and high FASN expression, and (4) low TC2N expression and low FASN expression. BC patients with high TC2N expression and low FASN expression exhibited the best overall survival rates, while those with low TC2N expression and high FASN expression showed the poorest overall survival rates (Fig. 7F). Collectively, these results indicate that TC2N predicts favorable prognosis by controlling FASN protein expression to inhibit BC progression (Fig. 8).

## Discussion

Metastasis and tumor heterogeneity leads to the poor clinical outcome. Identification and functional characterization of new targets and mechanisms contributing to the progression of BC represents, therefore, a major research priority. Our previous study preliminary inquired the anti-cancer role of TC2N in BC [18], a novel tandem C2 domain-containing protein turned into oncogene in many human cancers [12]. Despite the emerging roles of TC2N in tumor suppression, only very little is known about its function and underlying mechanism. In current study, our speculation of the inhibitory effect of TC2N on metastasis and CSC comes from the link between TC2N expression and metastatic status and tumor differentiating degree of patients. Parallelly, further gain and loss of function analysis testified that TC2N is crucial for suppressing the malignant phenotype of BC cells in vitro and in vivo, including metastasis and self-renewal. Regarding the mechanism of the tumor-suppressing role of TC2N, we show that TC2N restrains fatty acid synthesis by regulating FASN at protein level. These results were verified in clinical specimens, demonstrating for the first time that patients with breast tumors expressing high levels of TC2N have significantly lower FASN expression and fatty deposits than patients with tumors expressing lower TC2N levels. Meanwhile, we noted that TC2N staining was shown to accumulate outside the cellular compartments (Fig. 4F and G). Considering the important role of the C2 domain in regulating corresponding protein’s exocytosis function [36, 37], we regarded TC2N as a secreted protein that could translocate to extracellular microenvironment and affect tumor cell phenotype. Then, TC2N knockout mice (K/K: homozygous deletion of TC2N; K/W: heterozygous deletion of TC2N; W/W: wild type of TC2N) identified by RT-PCR (Additional file 10: Fig. S4A) was used to preliminary inquiry our speculation. E0771, a mouse BC cell line with high malignant potential [38], was selected and subcutaneously injected into the fifth mammary fat pad of TC2N K/K and TC2N W/W female mice to produce tumor models. Compared with no tumor formation in TC2N W/W mice, breast lumps were produced in 3 out of 4 TC2N K/K mice at 21 days after injection (Additional file 10: Fig. S4B). These data suggested that TC2N knockout in breast make the tumor more prone to growth. Based on above results, we theoretically support that TC2N may be secreted extracellularly and play a regulatory role in the tumor microenvironment. This aspect should, therefore, be elucidated in future studies as it could potentially represent an important target for the prevention and therapy.

Due to TC2N expression showed differences between each BC molecular subtype, we further evaluated the possible correlation between TC2N and FASN expression in the four cancer subtypes. Inconsistently, the correlation between TC2N and FASN level did not reach statistical significance in each molecular subtype of BC separately, does that mean the regulatory effort of TC2N on FASN expression only occur across on some BC molecular subtypes? In the aforementioned results, overexpressed TC2N repressed FASN in two BC cell lines, MCF7 (luminal A subtype) and M231 (basal-like subtype) (Fig. 4I). And now, we detected FASN expression change after TC2N overexpression in other two BC cell lines with different molecular subtypes, including SKBR3 for HER2^+^ subtype and BT474 for luminal B subtype. As expected, TC2N could inhibit FASN protein expression in these cell lines (Additional file 9: Fig. S3E), suggesting the effect of TC2N on FASN expression may be independent of BC molecular subtype, and the expression of TC2N did not show statistical correlation with FASN expression in all of the subtypes of BC probably because the sample size of each molecular subtype of BC is too small to analyze. The overrepresentation of FASN in HER2^+^ BC is well known [39]. Despite the average value of FASN was highest in HER2^+^ subtype among these subtypes, its expression had no significant difference among the four subtypes in our samples, indicating our limited sample size from a side.

The tumor suppressor, phosphatase and tensin homolog deleted on chromosome ten (PTEN) was first identified in 1997 [40, 41]. Through its lipid phosphatase activity, PTEN control a variety of tumor cell progressions, such as proliferation, apoptosis, invasion and metastasis by regulating PI3K/Akt/mTOR, FAK, Raf/MEK/ERK signaling pathway [42]. In BC, the loss of PTEN expression associated with large tumor sizes, lymph node metastasis, and an aggressive triple-negative phenotype [43]. However, PTEN loss-of-function in tumorigenesis is still poorly understood. Xie et al. provide a series of data to prove that PTEN is a neddylation substrate. More importantly, the high glucose level led to a clear increase in PTEN neddylation, this neddylated PTEN moves to the nucleus and acts as a cancer-assistant. by blocking TRIM21 induced FASN degradation in BC [33]. Similarly, Hu et al*.* demonstrated that the environment with sufficient nutrients leads to a polyubiquitination of PTEN, this post-translational modification alters its phosphatase activity and shows a stronger effect on tumor promotion [44]. These data uncovered a previously unidentified dark side of PTEN in cancer. In present study, we elucidated that TC2N facilitated the instability of FASN protein by regulating this “evil” PTEN. Mechanism-wise, TC2N blocked neddylated PTEN-mediated FASN stabilization by two kinds of mechanisms. In the cell nucleus, nuclear TC2N prevented PTEN but promoted TRIM21 from interacting with FASN to influence its protein stability. Oppositely, after the deletion of C2B domain, which represents the nuclear localization signal of TC2N, resulted in loss of FASN-binding function. Theoretically, the interaction between PTEN and FASN is supposed to fully rather than partly recover without the existence of TC2N in the nucleus. Combining that only a part of FASN level was recused after C2B domain deletion, we therefore speculated that cytoplasmic TC2N also exerts a tumor-suppressive function in BC. According to the IP assays, cytoplasmic TC2N contribute to the inhibition of PTEN neddylation. Previous studies have indicated that neddylation plays a key role in certain nuclear processes [45, 46]. The nuclear translocation of PTEN is required for neddylated PTEN generation and further FASN stabilization [33]. Therefore, there is only one possible way by which cytoplasmic TC2N inhibits PTEN neddylation, that is TC2N influence PTEN localization. We detected a decreased nuclear localization of PTEN in TC2N-overexpressed BC cells, while the expression of PTEN was unchanged. There are two possible explanations for the translocation of PTEN when TC2N is present: (1) TC2N inhibits PTEN nuclear import (2) TC2N promotes PTEN nuclear export. Nucleocytoplasmic transport is a complex process dependent on importin and exportin [47]. Here, we found that TC2N interacts with two nuclear import factors, which binds to and promotes the nuclear import of PTEN. It supports the concept that TC2N decreased PTEN nuclear distribution via interference with its nuclear import. Indeed, TC2N competed with PTEN for binding these importins and followed by decrease in the neddylation level of PTEN. However, we were unable to further identify which domains required for the TC2N-import protein interaction. This aspect should, therefore, be elucidated in future studies. To the best of our knowledge, the current study demonstrates for the first time that TC2N overexpression inhibits PTEN neddylation. Given the other post-translational modifications, such as SUMOylation and phosphorylation contribute to nucleocytoplasmic regulation of PTEN [33, 48–50], more investigations are required to address whether these modifications plays a key role in TC2N-mediated PTEN nuclear translocation in future studies. Up to now, increasing evidence supports the concept that PTEN stays in the cytoplasm to exerts its inhibitory effects on the PI3K-Akt signaling pathway [51–53], whereas the nuclear localization of neddylated PTEN exerts the opposite function to indulge PI3K-Akt signaling. From another point of view, TC2N appears to gain a molecular mechanism in the block of PI3K-Akt signaling pathway to support our previous work [18].

Increasing evidence indicates that elevated levels of FASN are a hallmark and are considered as a potential therapeutic target for BC [29, 54, 55]. Regrettably, many FASN inhibitors have failed in the clinical trial phase to date, largely because of poor solubility and severe side effect [56–61]. Here, TC2N showed a potent inhibitory effect on FASN-mediated metastatic and stem-like characteristics of BC, which means that it might be a potential inhibitor for FASN and is also worth exploring its clinical application value. Prior to this, we first discuss whether the TC2N/FASN axis-mediated tumor progression is BC subtype-dependent? Xie et al.’s study showed that the levels of neddylated PTEN were elevated and its nuclear expression positively correlated with FASN expression in all of the subtypes of BC. Then, combining their results with our results about TC2N inhibits FASN in all BC cell lines with different subtypes, we here from a side explains that the existence of TC2N is probably used for blocking neddylated PTEN/FASN axis-mediated tumor progression in all the four subtypes of BC. Of course, this assumption needs to be clarified in the future.

## Conclusions

Taken together, TC2N for the first time was revealed to inhibit metastasis, stem-like properties and fatty acid synthesis. Our findings suggested that both nuclear and cytoplasmic TC2N can lead to FASN degradation by orchestrating neddylated PTEN via an independent mechanism. This research provides a novel explanation of how TC2N suppresses tumor progression and suggests that TC2N is a potential therapeutic strategy for BC treatment.

### Supplementary Information

**Additional file 1: Table S1.** Clinicopathologic characteristics of BC patients.

**Additional file 2: Data S1.** The code for analyzing the expression correlation between TC2N and other genes or proteins.

**Additional file 3: Data S2.** The co-expressed genes or proteins of TC2N in BC.

**Additional file 4: Data S3.** Reactome Pathway enrichment analysis by GSEA.

**Additional file 5: Data S4.** UHPLC-MS/MS data of fatty acid composition (μg/mg).

**Additional file 6: Table S2.** Primer sequences for qRT-PCR assays.

**Additional file 7: Figure S1.** The analysis of the relationship between TC2N expression and biological processes and clinical outcome. A. 26 GEO datasets identified the association between TC2N expression and metastasis and differentiation-related processes. B. Kaplan–Meier survival analysis of TC2N expression with OS in Kaplan Meier plotter database. C. Kaplan–Meier survival analysis of TC2N expression with OS in GSE25307 dataset.

**Additional file 8: Figure S2.** TC2N interdicts BC progression. A. Cellular morphology of adherent BC cells compared to spheroid‐forming BC cells. WB analysis of TC2N expression in adherent and spheroid‐forming BC cells (Right). B and C. The expression of CSCs marker ALDH (FITC) in BC cells with ectopic expression of TC2N was analyzed by flow cytometry. Mean ± SEM. (n = 3). The P value was measured with Student’s t-tests. *P < 0.05, **P < 0.01, ***P < 0.001. D and E. Soft agar colony formation ability of spheroid‐forming BC cells with ectopic expression of TC2N. Mean ± SEM. (n = 3). The P value was measured with Student’s t-tests. **P < 0.01. F. Fractions of EdU-positive M231 cells were detected by flow cytometry. Mean ± SEM. (n = 3). The P value was measured with Student’s t-tests. ***P < 0.001.

**Additional file 9: Figure S3.** TC2N involves in regulating lipid metabolism and FASN expression in BC. A-D. The public databases identified the association between TC2N expression and lipid metabolism. E. WB revealed that TC2N decreases the protein expression of FASN in SKBR3 and BT474 cells.

**Additional file 10: Figure S4.** The knockout of TC2N suppressed the in situ growth of breast cancer cells. A. Genotype identification of TC2N engineered mice. MK: DNA marker; NC: Negative control. WT with but KO without band represent TC2N W/W mouse; Both of WT and KO with band represent TC2N K/W mouse; KO with but WT without band represent TC2N K/K mouse. B. The growth of E0771 cells in TC2N + / + and TC2N-/- mice (n = 4). White arrows indicate the tumor masses.

## Data Availability

The protein expression data of BC patients were extracted from Clinical Proteomic Tumor Analysis Consortium (CPTAC) database, and the mRNA-Seq and RNA expression profiles were downloaded from The Cancer Genome Atlas (TCGA) and GEO databases. The co-expressed proteins were functionally annotated by Gene Ontology (GO) and Reactome pathway analyses, and the significant biological processes were identified. The primary experiment data and material can be obtained from the correspondence with reasonable require.

## Abbreviations

BC: Breast cancer
M231: MDA-MB-231
TC2N: Tandem C2 domains, nuclear
FASN: Fatty acid synthase
TRIM21: Tripartite motif containing 21
PTEN: Phosphatase and tensin homolog
IHC: Immunohistochemistry
TMA: Tissue microarray
WB: Western blot
IF: Immunofluorescence
co-IP: Co-immunoprecipitation
