# Supplementary material for: TC2N inhibits distant metastasis and stemness of breast cancer via blocking fatty acid synthesis
Source: J Transl Med. 2024 Jan 2;22:6. doi: 10.1186/s12967-023-04721-3 (PMC10763294; doi:10.1186/s12967-023-04721-3)
Supplement: Supplementary file 1 — Additional file 1: Table S1. Clinicopathologic characteristics of BC patients. [file 12967_2023_4721_MOESM1_ESM.docx]

**Table S1.** Clinicopathologic characteristics of BC patients

|  | | |
| --- | --- | --- |
| **Characteristic** |  | **Number of Patients** |
| Patients |  | 212 |
| Age (years) |  | 24-80, median=48 |
| Clinical stage (AJCC) |  |  |
| Ⅰ |  | 20 |
| Ⅱ |  | 80 |
| Ⅲ |  | 65 |
| IV |  | 47 |
| Histological grade |  |  |
| 1 |  | 31 |
| 2 |  | 130 |
| 3 |  | 51 |
| Depth of tumor invasion |  |  |
| T_1_ |  | 44 |
| T_2_ |  | 79 |
| T_3_ |  | 75 |
| T_4_ |  | 14 |
| Lymph node metastasis |  |  |
| N_0_ |  | 65 |
| N_1_ |  | 67 |
| N_2_ |  | 45 |
| N_3_ |  | 35 |
| Distant metastasis |  |  |
| M_0_ |  | 165 |
| M_1_ |  | 47 |
| Primary site |  |  |
| Left breast |  | 137 |
| Right breast |  | 75 |
| Disease type |  |  |
| Ductal BC |  | 181 |
| Lobular BC |  | 31 |
| Molecular subtype |  |  |
| Luminal A |  | 59 |
| Luminal B |  | 54 |
| HER2+ |  | 40 |
| Basal-like |  | 59 |
| Clinical stage, tumor-nodes-metastasis, based on the American Joint Committee on Cancer/International Union Against Cancer Staging Manual (7th edition, 2009) | | |
|  |  |  |
|  |  |  |
