# Supplementary material for: TC2N inhibits distant metastasis and stemness of breast cancer via blocking fatty acid synthesis
Source: J Transl Med. 2024 Jan 2;22:6. doi: 10.1186/s12967-023-04721-3 (PMC10763294; doi:10.1186/s12967-023-04721-3)
Supplement: Supplementary file 6 — Additional file 6: Table S2. Primer sequences for qRT-PCR assays. [file 12967_2023_4721_MOESM6_ESM.docx]

**Table S2. Primer sequences for qRT-PCR assays**

| **Gene symbol** | **Forward Sequence** | **Reverse Sequence** |
| --- | --- | --- |
| Primers for qPCR of human target genes in knockdown experiments | | |
| TC2N | TGGCTGTACTGAGGATTATTTGC | TGTGAAGGAGTTTCTTGTGTCC |
| FASN | AACTCCTTGGCGGAAGAG | TAGGACCCCGTGGAATGTCA |
| ACTIN | CCACGAAACTACCTTCAACTCC | GTGATCTCCTTCTGCATCCTGT |
