# Supplementary figures and images for: TC2N inhibits distant metastasis and stemness of breast cancer via blocking fatty acid synthesis
Source: J Transl Med. 2024 Jan 2;22:6. doi: 10.1186/s12967-023-04721-3 (PMC10763294; doi:10.1186/s12967-023-04721-3)

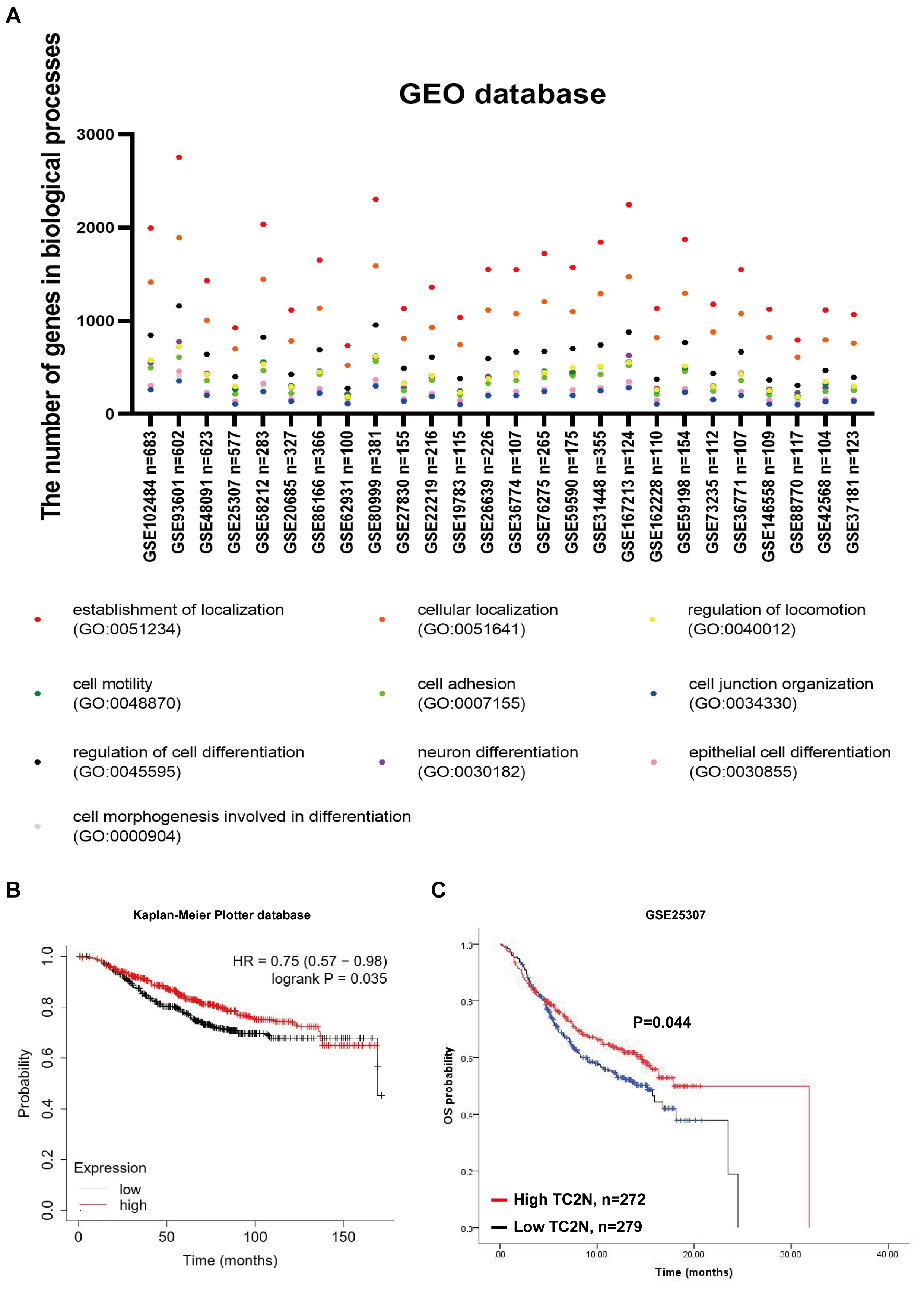

Supplement: Supplementary file 7 — Additional file 7: Figure S1. The analysis of the relationship between TC2N expression and biological processes and clinical outcome. A. 26 GEO datasets identified the association between TC2N expression and metastasis and differentiation-related processes. B. Kaplan–Meier survival analysis of TC2N expression with OS in Kaplan Meier plotter database. C. Kaplan–Meier survival analysis of TC2N expression with OS in GSE25307 dataset. [file 12967_2023_4721_MOESM7_ESM.tif]

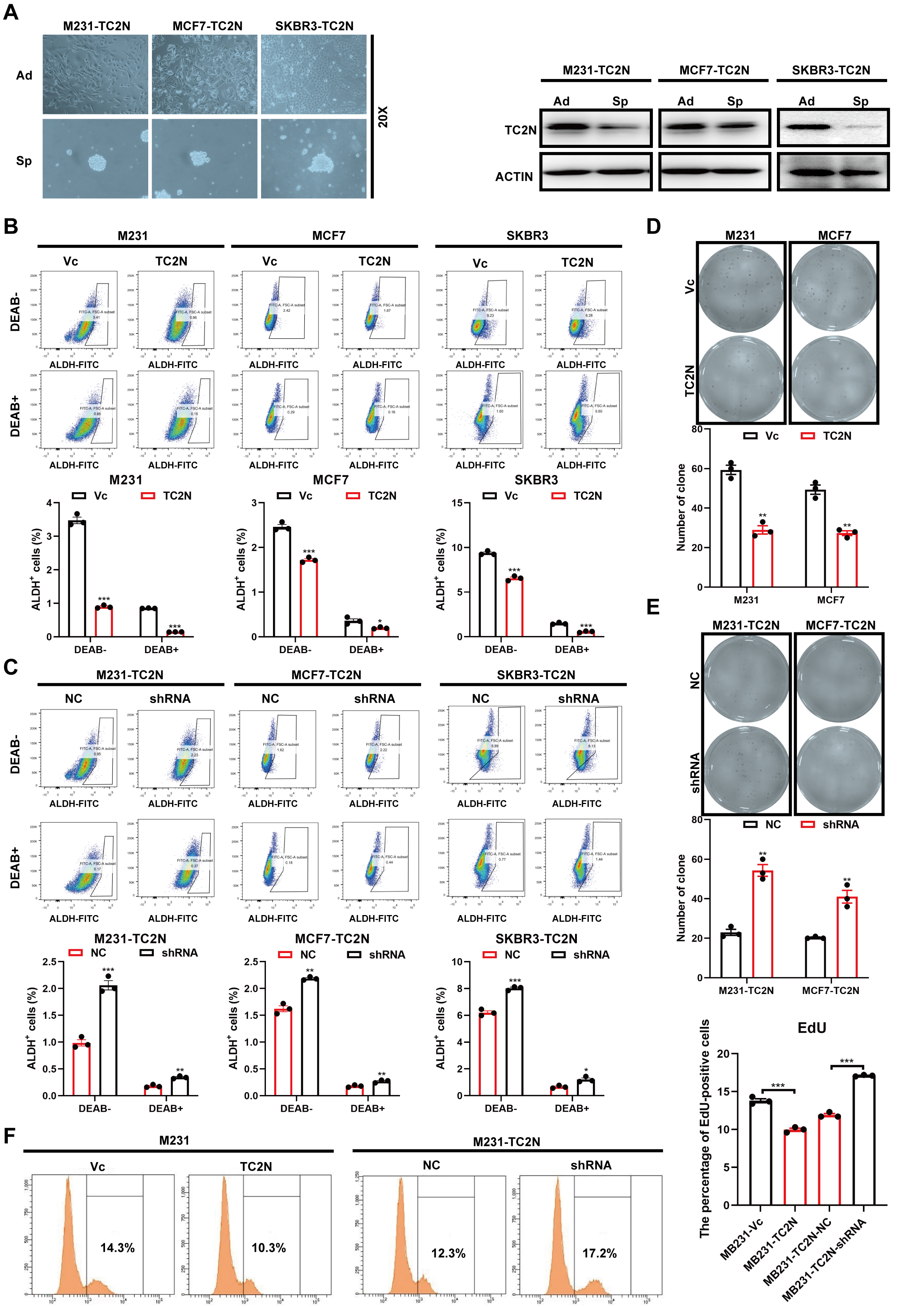

Supplement: Supplementary file 8 — Additional file 8: Figure S2. TC2N interdicts BC progression. A. Cellular morphology of adherent BC cells compared to spheroid‐forming BC cells. WB analysis of TC2N expression in adherent and spheroid‐forming BC cells (Right). B and C. The expression of CSCs marker ALDH (FITC) in BC cells with ectopic expression of TC2N was analyzed by flow cytometry. Mean ± SEM. (n = 3). The P value was measured with Student’s t-tests. *P < 0.05, **P < 0.01, ***P < 0.001. D and E. Soft agar colony formation ability of spheroid‐forming BC cells with ectopic expression of TC2N. Mean ± SEM. (n = 3). The P value was measured with Student’s t-tests. **P < 0.01. F. Fractions of EdU-positive M231 cells were detected by flow cytometry. Mean ± SEM. (n = 3). The P value was measured with Student’s t-tests. ***P < 0.001. [file 12967_2023_4721_MOESM8_ESM.tif]

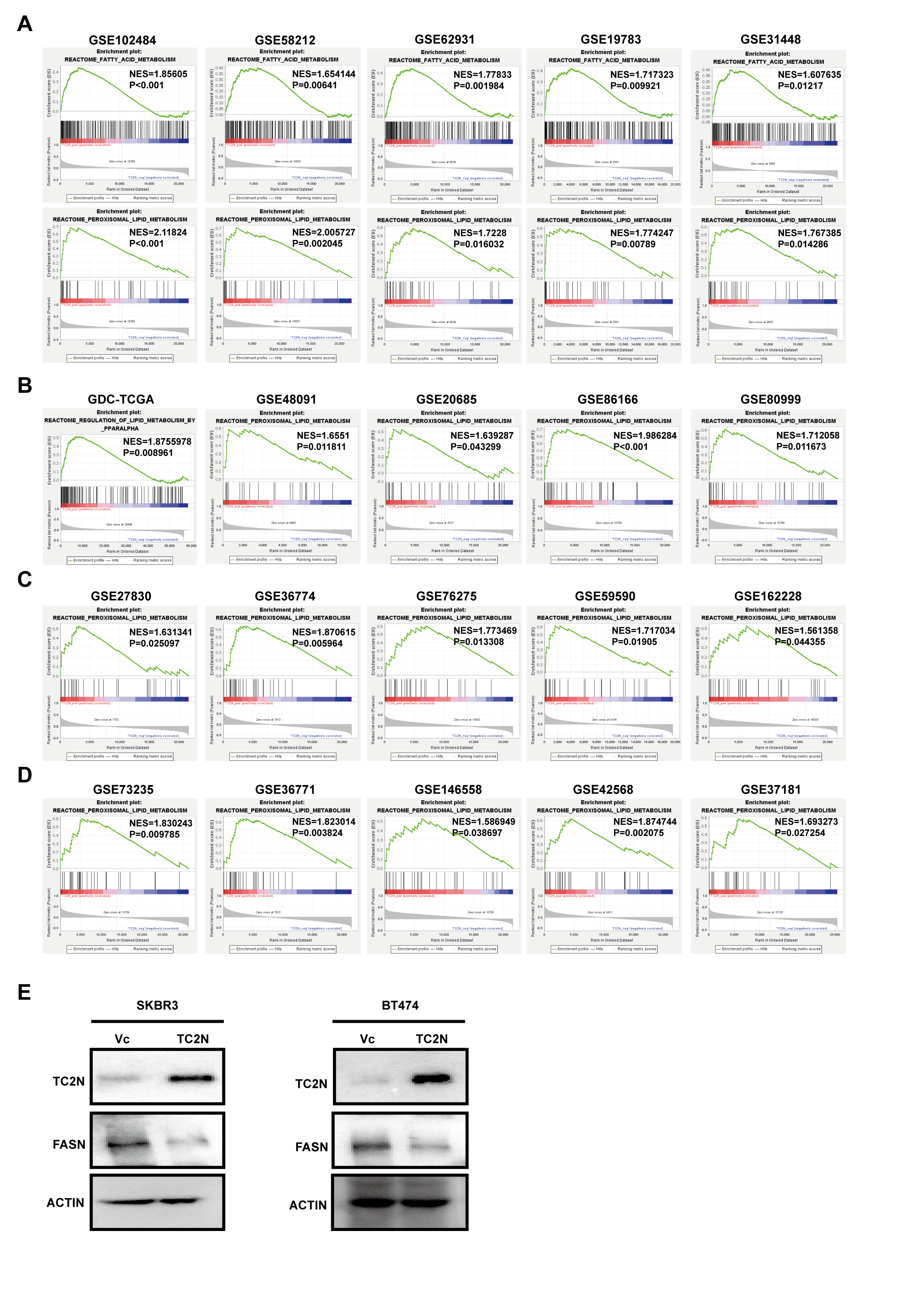

Supplement: Supplementary file 9 — Additional file 9: Figure S3. TC2N involves in regulating lipid metabolism and FASN expression in BC. A-D. The public databases identified the association between TC2N expression and lipid metabolism. E. WB revealed that TC2N decreases the protein expression of FASN in SKBR3 and BT474 cells. [file 12967_2023_4721_MOESM9_ESM.tif]

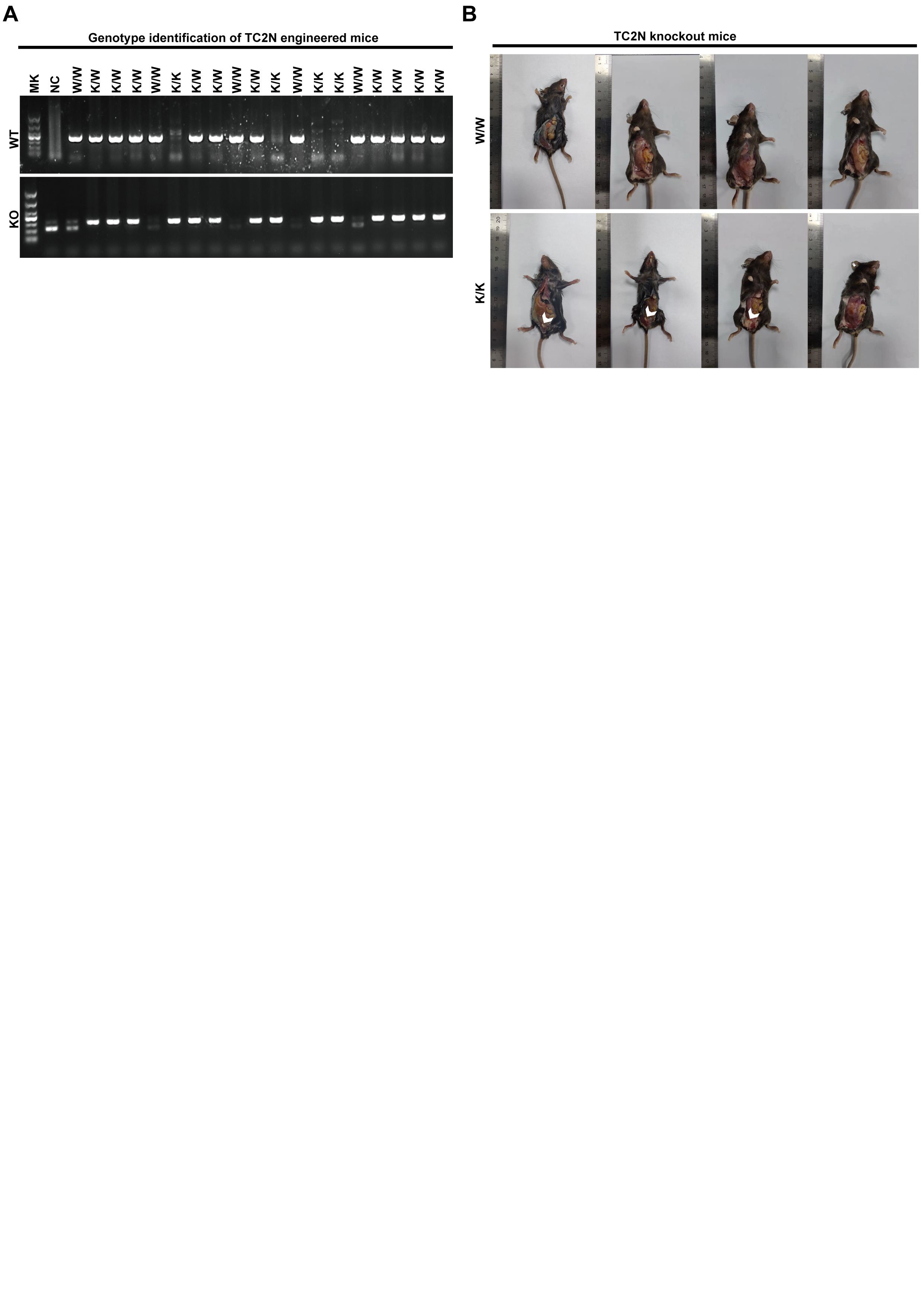

Supplement: Supplementary file 10 — Additional file 10: Figure S4. The knockout of TC2N suppressed the in situ growth of breast cancer cells. A. Genotype identification of TC2N engineered mice. MK: DNA marker; NC: Negative control. WT with but KO without band represent TC2N W/W mouse; Both of WT and KO with band represent TC2N K/W mouse; KO with but WT without band represent TC2N K/K mouse. B. The growth of E0771 cells in TC2N + / + and TC2N-/- mice (n = 4). White arrows indicate the tumor masses. [file 12967_2023_4721_MOESM10_ESM.tif]
